# Specific Metabolic Markers Are Associated with Future Waist-Gaining Phenotype in Women

**DOI:** 10.1371/journal.pone.0157733

**Published:** 2016-06-20

**Authors:** Benedikt Merz, Ute Nöthlings, Simone Wahl, Marjolein Haftenberger, Anja Schienkiewitz, Jerzy Adamski, Karsten Suhre, Rui Wang-Sattler, Harald Grallert, Barbara Thorand, Tobias Pischon, Ursula Bachlechner, Anna Floegel, Annette Peters, Heiner Boeing

**Affiliations:** 1 Department of Nutrition and Food Sciences, University of Bonn, Bonn, Germany; 2 Research Unit of Molecular Epidemiology, Helmholtz Zentrum München – German Research Center for Environmental Health, Neuherberg, Germany; 3 Institute of Epidemiology II, Helmholtz Zentrum München – German Research Center for Environmental Health, Neuherberg, Germany; 4 German Center for Diabetes Research (DZD), Neuherberg, Germany; 5 Robert Koch-Institut, Department of Epidemiology and Health Monitoring, Berlin, Germany; 6 Institute of Experimental Genetics, Genome Analysis Center, Helmholtz Zentrum München, German Research Center for Environmental Health, Neuherberg, Germany; 7 Institute of Bioinformatics and Systems Biology, Helmholtz Zentrum München, German Research Center for Environmental Health, Neuherberg, Germany; 8 Department of Physiology and Biophysics, Weill Cornell Medical College in Qatar, Doha, Qatar; 9 Molecular Epidemiology Research Group, Max Delbrück Center for Molecular Medicine (MDC), Berlin, Germany; 10 Department of Epidemiology, German Institute of Human Nutrition, Potsdam-Rehbruecke, Germany; Johns Hopkins Bloomberg School of Public Health, UNITED STATES

## Abstract

**Objective:**

Our study aims to identify metabolic markers associated with either a gain in abdominal (measured by waist circumference) or peripheral (measured by hip circumference) body fat mass.

**Methods:**

Data of 4 126 weight-gaining adults (18–75 years) from three population-based, prospective German cohort studies (EPIC, KORA, DEGS) were analysed regarding a waist-gaining (WG) or hip-gaining phenotype (HG). The phenotypes were obtained by calculating the differences of annual changes in waist minus hip circumference. The difference was displayed for all cohorts. The highest 10% of this difference were defined as WG whereas the lowest 10% were defined as HG. A total of 121 concordant metabolite measurements were conducted using Biocrates Absolute*IDQ*^®^ kits in EPIC and KORA. Sex-specific associations with metabolite concentration as independent and phenotype as the dependent variable adjusted for confounders were calculated. The Benjamini-Hochberg method was used to correct for multiple testing.

**Results:**

Across studies both sexes gained on average more waist than hip circumference. We could identify 12 metabolites as being associated with the WG (n = 8) or HG (n = 4) in men, but none were significant after correction for multiple testing; 45 metabolites were associated with the WG (n = 41) or HG (n = 4) in women. For WG, n = 21 metabolites remained significant after correction for multiple testing. Respective odds ratios (OR) ranged from 0.66 to 0.73 for tryptophan, the diacyl-phosphatidylcholines (PC) C32:3, C36:0, C38:0, C38:1, C42:2, C42:5, the acyl-alkyl-PCs C32:2, C34:0, C36:0, C36:1, C36:2, C38:0, C38:2, C40:1, C40:2, C40:5, C40:6, 42:2, C42:3 and lyso-PC C17:0.

**Conclusion:**

Both weight-gaining men and women showed a clear tendency to gain more abdominal than peripheral fat. Gain of abdominal fat seems to be related to an initial metabolic state reflected by low concentrations of specific metabolites, at least in women. Thus, higher levels of specific PCs may play a protective role in gaining waist circumference.

## Introduction

Overweight and obesity are major public health problems in Germany. More than two-thirds of the male and more than half of the female population were classified as overweight in a representative nationwide survey conducted between 2008 and 2011 [1]. Increased fat mass is associated with the risk of hypertension and an increased risk for type 2 diabetes, cardiovascular disease (CVD) and several types of cancer [2–4]. Body weight gain during adulthood independent of initial adult body mass index (BMI) was reported to be an independent risk factor for diabetes [5, 6] and is in general accompanied by an increase in total body fat.

The distribution of body fat has been shown to be independently associated with chronic diseases [7–9] and a distinction should be made between abdominal and gluteofemoral phenotypes. Measurements of waist and hip circumference have been proven useful to assess body fat distribution [10, 11]. Waist circumference has been shown to be a good measure of visceral adipose tissue and abdominal obesity [12, 13]. Visceral fat, which surrounds the inner organs and increases with abdominal fat accumulation, is accompanied by unfavourable metabolic changes due to its role as an endocrine organ [14, 15]. In contrast, larger hip circumference represents gluteofemoral and peripheral adipose tissue and therefore subcutaneous, non-visceral fat [11]. Some studies even reported inverse associations of markers of these peripheral fat compartments regarding CVD and mortality [7, 16–19]. Evidence exists that larger hip circumference attenuates risk associations based on large waist circumference [8, 19], thus not only the waist to hip ratio (WHR)–an established anthropometric marker of risk—but also the waist–hip difference could be a very interesting risk marker.

We already know some determinants of body fat distribution such as age, sex and genetics [11, 20–22]; in particular gene-sex interactions are responsible for the two distinct phenotypes of abdominal and gluteofemoral body shape [22, 23]. However, it is not well known whether metabolic status further favours the process of fat deposition at a specific site.

Metabolomics aims to profile low-molecular-weight metabolites and is a promising tool in the assessment of metabolic status [24, 25]. Thus we applied targeted metabolomics to investigate whether there is a link between these metabolic profiles and the two distinct types of body fat deposition during weight gain.

## Materials and Methods

### Study design

The full analysis is based on data from the European Prospective Investigation into Cancer and Nutrition Potsdam Study (EPIC, 1994–2008), and the Cooperative Health Research in the Region of Augsburg Study, (KORA, 1999–2008). Detailed information on the background of the studies and the recruitment procedures have been described previously [13, 15, 26].

Data of the German Health Interview and Examination Survey for Adults (DEGS, 1997–2011) [13] were additionally used to provide nationwide data for Germany regarding the difference between waist and hip circumference since EPIC and KORA represent populations from either Brandenburg or the region of Augsburg (Bavaria). Each study was conducted after approval of the respective local ethic committees (the medical association of the state of Brandenburg, Germany; Bavarian Medical Association; Charité-Universitätsmedizin Berlin) and according to the guidelines of the Declaration of Helsinki. All participants gave written informed consent prior to study participation.

### Study population

The EPIC cohort consists of 27 548 participants mainly in the aged 35–65 (women) and 40–65 (men) at recruitment, of which a subcohort of 2 500 participants was randomly drawn for specific biomarker measurements using a case-cohort design [27]. The study population was biannually contacted by mail for follow-up information. The KORA S4/F4 cohort consists of 4 261 participants, of which 3 080 individuals also participated in a follow-up examination [28]. In a subsample of 1 614 participants aged 54 to 75, targeted metabolomics measurements were performed [29]. The DEGS is part of the health monitoring conducted by the Robert Koch-Institut. Participants from the survey in 1998 (GNHIES98, N = 7 124) were reinvited to participate in 2008–2011. 3 959 participants from GNHIES98 (response rate 62%), of whom 914 were interviewed only and 3 045 were both interviewed and examined [30]. Therefore, our study included a total of 7 159 participants (EPIC: 2 500, KORA: 1 614, DEGS: 3 045). Of these participants, 829 individuals with missing information for variables of interest at baseline or follow-up (EPIC: 299, KORA: 496, DEGS: 34) and 24 pregnant women (DEGS) were excluded. Because of our interest in specific weight gain phenotypes, 2 193 individuals without body weight gain in the follow-up period (EPIC: 557, KORA: 526, DEGS: 1 110) were also excluded resulting in a final analytical study sample of 4 126 individuals (EPIC: 1 644, KORA: 592, DEGS: 1 890), 1 802 men and 2 324 women.

### Anthropometric assessment

For the baseline examination, participants were invited to the study centres and examined by trained staff according to study-specific standardized procedures in a standing position using a non-elastic flexible tape. In EPIC, all baseline measures were performed in underwear without shoes, in KORA and DEGS measures were performed in light clothing without shoes. Waist circumference (in cm) was measured at the midpoint (EPIC [31]) and the narrowest point (KORA, DEGS [32]) between the lowest rib and the superior border of the iliac crest. Hip circumference (in cm) was measured at the most sweeping point of the buttocks horizontally around the body in EPIC [30] and at the widest protrusion of the gluteal region between the superior border of the iliac crest and crotch in KORA and DEGS [32].

For the follow-up examination in EPIC, information on body weight, waist and hip circumference was collected as a self-report. Participants received a letter with a standardized non-elastic flexible tape and written instructions [33]. To reduce bias due to measurement error and underreporting, self-reported values were corrected using equations developed for EPIC self-measurements [34, 35]. For the follow-up examination in study centres of DEGS, a slight modification in standardized procedures was made measuring participants in underwear instead of light clothing at baseline [13]. In KORA, waist and hip circumference was measured according to the same protocol as at baseline.

### Metabolomics measurement

For EPIC and KORA, serum samples were collected while participants attended the study centre for baseline examination. For appointments before noon, participants were informed about the blood sampling and advised not to eat anything before their examination [26]. Individuals with an overnight fast of more than 8 hours were categorized as fasting, all other as non-fasting. Serum concentrations of metabolites were determined using the Absolute*IDQ*^®^ p150 kit in EPIC and p180 kit in KORA (Biocrates Life Sciences AG, Innsbruck, Austria) in the Genome Analysis Center at the Helmholtz Zentrum München using the flow injection analysis tandem mass spectrometry (FIA-MS/MS) technique [36]. Sample preparation was done according to the manufacturer’s protocol (Biocrates user’s manuals UM-P150 & UM-P180) and described in detail elsewhere [36, 37]. In brief, after centrifugation, 10 mL serum was inserted into a filter on a 96-well sandwich plate, which already contained stable isotope-labelled internal standards. Amino acids (AA) were derivated with 5% phenyl isothiocyanate reagent. Metabolites and internal standards were extracted with 5 mmol/L ammonium acetate in methanol. This solution was centrifuged through a filter membrane and diluted with mass spectrometry running solvent. Final extracts were analysed by FIA-MS/MS, and metabolites were quantified in mmol/L by appropriate internal standards. The method has been validated, and analytical specifications were provided in the Biocrates manuals [38]. A total of 163 metabolites (p150) and 186 metabolites (p180) were quantified, including AA, acylcarnitines (AC), different kinds of glycerophospholipids (lyso-, diacyl-, and acyl-alkyl-phosphatidylcholines (PC)), sphingomyelins (SM), hexoses (sum of six-carbon monosaccharides without distinction of isomers) and biogenic amines (p180 only). Lipid side chains were abbreviated as Cx:y with x referring to the number of carbons in the side chain and y to the number of double bonds. In addition, PCs were differentiated based on their type of bond to glycerol with ‘a’ for an acyl and ‘e’ for an alkyl bond. AA were presented according to three-letter abbreviations. Serum or reference samples were measured multiple times to get within-plate or between-plate variance, respectively. The standard deviation for those measures was divided by the mean value of the data set for each metabolite to obtain the coefficient of variation (CV). For EPIC, metabolites below the limit of detection (n = 30) and with a CV greater than 50% (n = 6) were excluded from the analysis [38]. For KORA, metabolites with more than 5% missing values or a mean CV across plates greater than 25% (n = 20) were excluded from the analysis [29]. Missing value imputation and outlier treatment in KORA has been previously described [29]. The overlap of both kits after study-specific quality control was 121 metabolites.

### Data analysis

Study-specific descriptive data is presented as mean and standard deviation (SD) for continuous and absolute and relative frequencies for categorical variables. Student’s t-test or sign test for continuous variables and Chi-square or Fisher’s exact test for categorical variables were applied to test differences between groups. Inclusion criterion of body weight gain was defined as body weight at follow-up (in kg) minus body weight at baseline greater than 0. According to the criteria of the World Health Organization (WHO) [39] BMI (in kg/m^2^) was calculated as body weight divided by height (in m) squared and individuals were classified as overweight (BMI ≥ 25 kg/m^2^), pre-obese (25 ≤ BMI < 30 kg/m^2^) and obese (BMI ≥ 30 kg/m^2^). Individuals exceeding waist circumferences of 102 cm (men) and 88 cm (women) were categorized as abdominally obese [40, 41].

For body weight, waist and hip circumference, average annual changes were calculated by subtracting the baseline measure from the follow-up measure divided by the individual follow-up time in years. The absolute difference of average annual changes in waist circumference minus hip circumference was calculated. Negative values identified individuals gaining more hip than waist circumference, positive values identified people gaining more waist than hip circumference. For percentage changes per year, the difference between follow-up measure and baseline measure was first divided by the baseline measure of interest and then divided by the individual follow-up time.

We tested the absolute waist-hip difference for differences between sexes using Student’s t-test. Furthermore, we investigated the relationship between waist-hip difference and average annual weight change using Pearson’s correlation coefficients for men and women separately. The nationwide study of DEGS was additionally standardized to the structure of the German population at 31.12.1997 [42].

About 10% of each sex in EPIC had a negative value in the waist-hip difference. Therefore, the lowest 10% of the waist-hip difference were defined as the hip-gaining phenotype (HG) (with a tendency of gaining more hip circumference) and the highest 10% were defined as the waist-gaining phenotype (WG). The remaining individuals formed the reference category. This categorization was applied for each sex separately. In order to increase comparability of results, all metabolite concentrations were standardized to a mean of 0 and a standard deviation of 1. Because body fat distribution as well as metabolite concentrations are different in men and women [11, 23], and some metabolites showed significant interaction effects with sex, analysis was performed for men and women separately. For each metabolite, sex-specific multiple logistic regression models with either WG or HG as the dependent and standardized metabolite concentration as the independent variable were fitted, adjusted for age at recruitment, baseline BMI, waist and hip circumference, smoking status (ever, former, current smoker) and prevalent or incident chronic diseases (including myocardial infarction, stroke, diabetes and all types of cancer).

Results of EPIC and KORA were then combined using fixed-effect meta-analyses with inverse-variance weighting. The combined estimate had to fulfil two criteria to be regarded as a true association: 1) single study estimates had to be consistent concerning effect direction; 2) combined estimate had to be significant after correction for multiple testing.

We performed sensitivity analyses to test the robustness of our findings in a population without pre-mentioned prevalent or incident diseases, a fasting population and for menopausal women. Subgroup analyses were performed for alcohol consumption (cut-off <20 g/d for men and <10 g/d for women), physical activity (cut-off <1 h/week), age (cut-off >55 years) and abdominal obesity (waist circumference men ≥102 cm, women ≥88 cm). Calculations in all studies were done using SAS release 9.4 (SAS Institute, Cary, NC, USA). Fixed-effect meta-analysis was performed using the statistical software ‘R’, version 3.1.2 [43] with the R package ‘meta’, version 4.1–0. To correct for multiple testing, the false discovery rate (FDR) was controlled at 0.05 using the Benjamini-Hochberg method [44].

## Results

### Characteristics of the study population

The majority of weight-gaining participants in the studies were categorized as overweight according to their BMI with women in EPIC having the lowest (25.1 ± 4.4 kg/m^2^) and women in KORA having the highest average baseline BMI (28.3 ± 4.6 kg/m^2^) (Table 1). In all studies, men had significantly higher initial body weight, waist circumference and WHR compared to women (all p <.0001). Average annual weight change was comparable between sexes in each study, ranging from 0.64 ± 0.57%/yr for men in KORA up to 0.95 ± 0.80%/yr for women in EPIC (all sex-differences not significant). Participants of KORA had the highest levels of waist circumference (women: 89.1 ± 10.9 cm, men: 100.1 ± 9.1 cm) and the highest percentage of abdominally obese people (women: 51%, men: 35%). Participants of EPIC had the lowest initial measures of body weight, waist and hip circumference but the highest rates of average annual changes in those body measures (women: 0.95 ± 0.80%/yr body weight gain, 1.48 ± 0.89%/yr waist and 0.54 ± 0.52%/yr hip changes; men: 0.77 ± 0.62%/yr body weight gain, 1.33 ± 0.77%/yr waist and 0.50 ± 0.43%/yr hip changes). The national survey (DEGS) showed the lowest rates of annual changes in waist circumference (women: 0.94 ± 0.78%/yr; men: 0.63 ± 0.67%/yr). In men there was even a tendency of losing hip circumference on average (-0.10 ± 0.36%/yr).

**Table 1:** Participant characteristics of weight-gaining individuals from DEGS, EPIC and KORA ^a^.

	DEGS^b^		EPIC		KORA	
Baseline characteristics	Women (n = 1 006)	Men (n = 884)	Women (n = 1 015)	Men (n = 629)	Women (n = 303)	Men (n = 289)
Age at recruitment (yr)	43.3 ±11.8	41.8 ± 12.8	48.2 ± 9.0	51.5 ± 8.0	62.8 ± 5.2	62.6 ± 5.2
Body weight (kg)	68.3 ± 12.9	83.2 ± 11.8	66.9 ± 12.3	81.1 ± 11.4	71.9 ± 12.0	84.0 ± 11.2
Waist circumference (cm)	82.0 ± 11.4	94.9 ± 10.6	79.3 ± 11.4	93.0 ± 9.6	89.1 ± 10.9	100.1 ± 9.1
Hip circumference (cm)	104.0 ± 10.0	104.8 ± 6.3	100.4 ± 8.6	99.6 ± 6.0	106.9 ± 9.1	104.7 ± 6.6
BMI (kg/m^2^)	25.5 ± 4.7	26.6 ± 3.5	25.1 ± 4.4	26.5 ± 3.5	28.3 ± 4.6	28.2 ± 3.5
WHR	0.79 ± 0.06	0.90 ± 0.07	0.79 ± 0.07	0.93 ± 0.06	0.83 ± 0.06	0.96 ± 0.05
Prevalence of abdominal obesity (%)^c^	28.1	24.6	20.3	18.4	51.5	35.3
Alcohol consumption (g/d)	4.6 ± 8.4	15.5 ± 19.4	8.6 ± 10.5	24.5 ± 27.6	7.0 ± 10.7	25.9 ± 26.6
**Averaged percentage changes per year**						
Weight (%/yr)	0.77 ± 0.61	0.66 ± 0.60	0.95 ± 0.80	0.77 ± 0.62	0.78 ± 0.79	0.64 ± 0.57
Waist circumference (%/yr)	0.94 ± 0.78	0.63 ± 0.67	1.48 ± 0.89	1.33 ± 0.77	0.98 ± 0.82	0.77 ± 0.61
Hip circumference (%/yr)	0.18 ± 0.43	-0.10 ± 0.36	0.54 ± 0.52	0.50 ± 0.43	0.44 ± 0.73	0.42 ± 0.48
**Smoking status**						
Never smoker	286 (28.4)	308 (34.8)	598 (58.9)	181 (28.8)	214 (70.6)	91 (31.5)
Former smoker	166 (16.5)	234 (26.5)	237 (23.3)	287 (45.6)	67 (22.1)	147 (50.9)
Current smoker	554 (55.1)	342 (38.6)	180 (17.7)	161 (25.6)	22 (7.3)	51 (17.7)
**Physical activity**						
< 1 h/week	594 (59.1)	501 (56.8)	611 (60.1)	408 (64.8)	157 (51.8)	170 (58.8)
1–2h/week	209 (20.8)	150 (16.9)	263 (25.9)	110 (17.5)	94 (31.0)	66 (22.8)
> 2h/week	203 (20.1)	233 (26.3)	142 (14.0)	111 (17.7)	52 (17.2)	53 (18.3)
**Prevalence of diseases** ^d^	76 (7.6)	69 (7.8)	151 (14.9)	124 (19.7)	67 (22.1)	92 (31.8)

BMI, body mass index; WHR, waist-to-hip ratio

^a^ Values are mean ± SD or n (%)

^b^ DEGS is standardized to the structure of the German population at 31.12.1997

^c^ Abdominal obesity defined as waist circumference >88 cm (women) / >102 cm (men)

^d^ Including myocardial infarction, stroke, diabetes and all types of cancer

### Waist-hip difference

**Fig 1:**
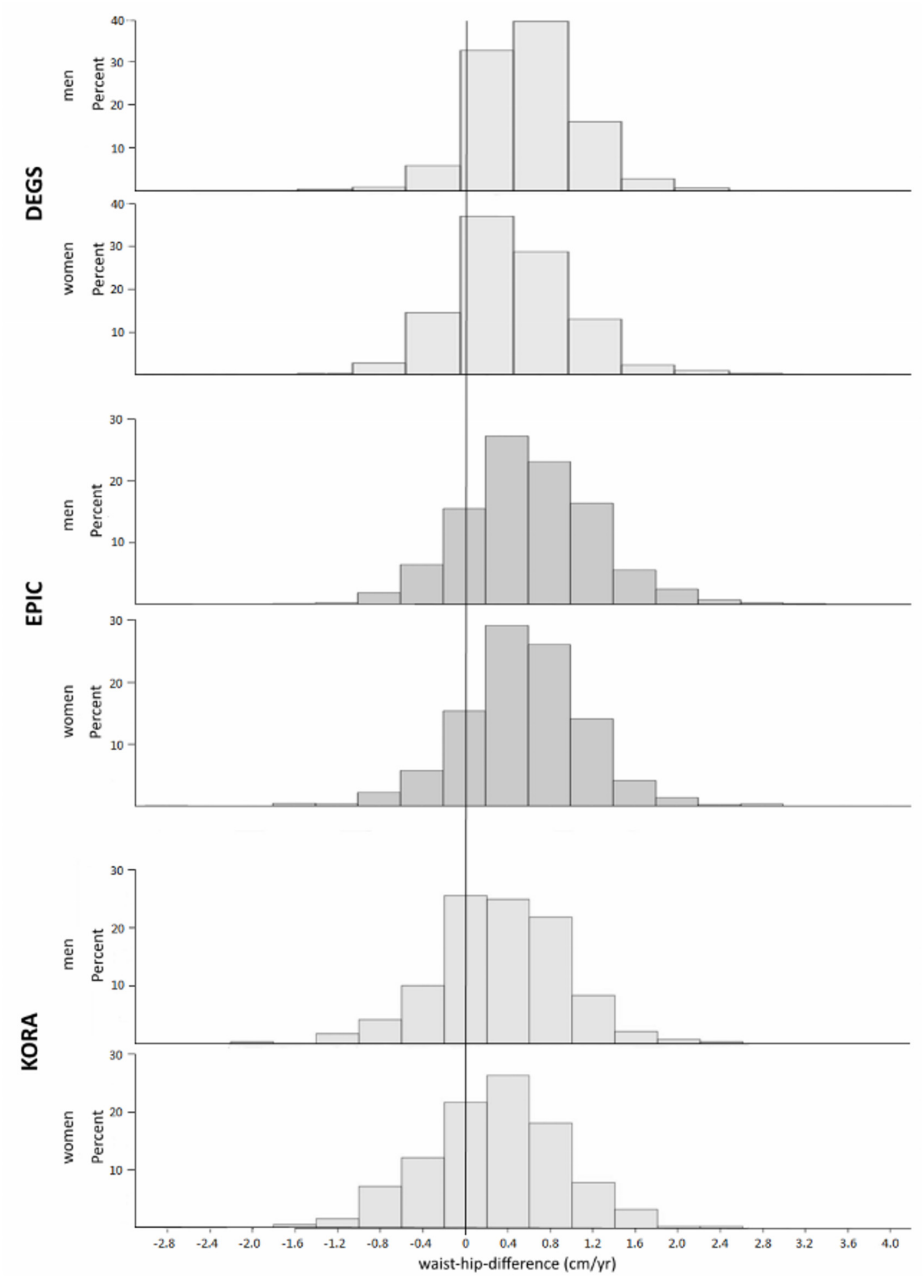
Distribution of waist-hip difference in weight-gaining individuals stratified by sex for each study. DEGS, German Health Interview and Examination Survey for adults; EPIC, European Prospective Investigation into Cancer and Nutrition Potsdam Study; KORA, Cooperative Health Research in the Region of Augsburg Study.

We observed a higher increase in waist circumference compared to hip circumference in all studies, independent of sex. The distribution of the waist-hip difference by study and sex is shown in Fig 1. The waist-hip difference was lowest in KORA (men: 0.33 ± 0.11 cm/yr; women: 0.29 ± 0.12 cm/yr) and highest in EPIC (men: 0.73 ± 0.25 cm/yr; women: 0.60 ± 0.14 cm/yr). There were significant differences between sexes in EPIC and DEGS (p <.0001) but not in KORA (p = 0.41). Correlation coefficients were consistently stronger between waist-hip difference and weight change in men (Table 2). Men in EPIC had the highest correlation coefficient (r = 0.41, p <.0001); in contrast, women in KORA had the lowest correlation coefficient with (r = 0.005, p = 0.927).

**Table 2:** Waist-hip difference by study and their correlation with weight change.

			waist-hip difference (cm/yr)		
	study	n	Mean	(95%-CI)	Pearson's r with weight change
**Men**	DEGS^a^	884	0.62	(0.59; 0.65)	0.27
	EPIC	629	0.73	(0.68; 0.78)	0.41
	KORA	289	0.33	(0.26; 0.40)	0.29
**Women**	DEGS^a^	1,006	0.47	(0.43; 0.50)	0.13
	EPIC	1,015	0.60	(0.56; 0.63)	0.10
	KORA	303	0.29	(0.22; 0.36)	0.005

^**a**^ standardized to structure of German population at 31.12.1997

We defined two different weight-gaining phenotypes by taking the two extreme deciles of the waist-hip difference in men and women: the HG in both sexes was characterized by highest initial body weight (EPIC: 76.7 ± 16.4 kg (women), 78.8 ± 10.6 kg (men); KORA: 76.7 ± 11.3 kg (women), 89.9 ± 13.4 kg (men)), highest initial waist and hip circumference (only exception hip circumference in men) but lowest rates of average annual body weight gain in men (EPIC: 0.41 ± 0.40%/yr; KORA: 0.49 ± 0.43%/yr). The WG showed highest rates of weight gain and on average younger individuals compared to the HG. In addition, we observed a tendency towards a larger increase in waist than hip circumference in the reference categories (S1 and S2 Tables).

### Phenotype associated metabolites

We observed metabolites to be more often associated with the WG compared to the HG. The meta-analytical combination of single study estimates revealed 8 metabolites in men and 41 metabolites in women associated with the WG; in contrast 4 metabolites for each sex were associated with the HG. In particular, PCs were associated with the WG. In women, those PCs showed consistent inverse associations to the relevant phenotypes; in men diacyl-PC C38:3 showed a positive association with the WG, the remaining three PCs showed inverse associations (Tables 3 and 4).

**Table 3:** Significant (p< 0.05) meta-analytic combined associations with corresponding confidence limits and p-values for both phenotypes in men.

	WG phenotype					HG phenotype			
Metabolite	OR	LCL	UCL	*p*	Metabolite	OR	LCL	UCL	*p*
**AC**					**AA**				
C18	1.39	1.09	1.79	0.0090	Orn	0.73	0.54	0.98	0.0345
**Diacyl-PC**					**AC**				
PC aa C38:3	1.33	1.03	1.70	0.0261	C5-OH (C3-DC-M)	1.33	1.04	1.69	0.0206
PC aa C40:2	0.69	0.52	0.91	0.0087	C7-DC	1.30	1.04	1.63	0.0226
PC aa C42:1	0.73	0.55	0.96	0.0241	**Diacyl-PC**				
**Acyl-alkyl-PC**					PC aa C38:1	0.74	0.56	0.98	0.0384
PC ae C32:2	0.71	0.53	0.95	0.0205					
**Lyso-PC**									
lysoPC a C17:0	0.74	0.55	0.99	0.0415					
**SM**									
SM C18:0	1.35	1.06	1.73	0.0168					
SM C18:1	1.41	1.10	1.80	0.0064					

a, acyl; AA, amino acids; AC, acylcarnitines; e, alkyl; LCL, lower 95% confidence limit; OR, odds ratio; PC, phosphatidylcholines; SM, sphingomyelin; UCL, upper 95% confidence limit

**Table 4:** Significant (p< 0.05) meta-analytic combined associations with corresponding confidence limits and p-values for both phenotypes in women.

	WG phenotype					HG phenotype			
Metabolite	OR	LCL	UCL	*p*	Metabolite	OR	LCL	UCL	*p*
**AA**					**Diacyl-PC**				
Trp	0.71	0.57	0.88	0.0021	PC aa C40:5	0.79	0.64	0.98	0.0351
**AC**					PC aa C40:6	0.79	0.63	0.99	0.0372
C16	0.77	0.62	0.96	0.0216	**Acyl-alkyl-PC**				
C18	0.79	0.63	0.98	0.0349	PC ae C32:2	1.25	1.00	1.56	0.0482
**Diacyl-PC**					**SM**				
PC aa C28:1	0.79	0.63	0.98	0.0316	SM C24:0	0.79	0.64	0.98	0.0333
PC aa C32:3	0.69	0.55	0.86	0.0010					
PC aa C36:0	0.70	0.56	0.88	0.0017					
PC aa C36:6	0.77	0.61	0.97	0.0262					
PC aa C38:0	0.69	0.56	0.86	0.0010					
PC aa C38:1	0.69	0.55	0.87	0.0016					
PC aa C38:6	0.76	0.61	0.94	0.0118					
PC aa C40:2	0.75	0.59	0.97	0.0288					
PC aa C40:3	0.76	0.60	0.95	0.0176					
PC aa C40:6	0.74	0.59	0.93	0.0097					
PC aa C42:0	0.79	0.64	0.98	0.0355					
PC aa C42:1	0.77	0.62	0.96	0.0194					
PC aa C42:2	0.72	0.56	0.91	0.0058					
PC aa C42:5	0.72	0.57	0.91	0.0055					
PC aa C42:6	0.76	0.61	0.95	0.0169					
**Acyl-alkyl-PC**									
PC ae C30:0	0.76	0.61	0.94	0.0114					
PC ae C30:2	0.74	0.59	0.94	0.0113					
PC ae C32:1	0.80	0.65	0.98	0.0317					
PC ae C32:2	0.73	0.59	0.90	0.0030					
PC ae C34:0	0.72	0.58	0.90	0.0039					
PC ae C34:1	0.79	0.64	0.98	0.0282					
PC ae C36:0	0.72	0.58	0.91	0.0053					
PC ae C36:1	0.73	0.59	0.91	0.0057					
PC ae C36:2	0.71	0.56	0.88	0.0026					
PC ae C38:0	0.68	0.54	0.85	0.0008					
PC ae C38:2	0.75	0.60	0.93	0.0082					
PC ae C38:6	0.78	0.63	0.96	0.0198					
PC ae C40:1	0.70	0.56	0.87	0.0011					
PC ae C40:2	0.73	0.58	0.91	0.0054					
PC ae C40:3	0.78	0.63	0.96	0.0215					
PC ae C40:4	0.77	0.61	0.96	0.0183					
PC ae C40:5	0.73	0.58	0.91	0.0057					
PC ae C40:6	0.66	0.52	0.82	0.0002					
PC ae C42:2	0.68	0.54	0.84	0.0004					
PC ae C42:3	0.67	0.53	0.83	0.0003					
**Lyso-PC**									
lysoPC a C17:0	0.73	0.58	0.91	0.0049					
lysoPC a C18:0	0.81	0.65	0.99	0.0403					
lysoPC a C18:1	0.79	0.63	0.99	0.0396					

a, acyl; AA, amino acids; AC, acylcarnitines; e, alkyl; LCL, lower 95% confidence limit; OR, odds ratio; PC, phosphatidylcholines; SM, sphingomyelin; UCL, upper 95% confidence limit

Diacyl-PCs C40:2, C42:1, acyl-alkyl-PC C32:2 and lyso-PC C17:0 were observed to be inversely associated with the WG in both sexes, whereas C18 showed an inverse association with the WG in women but a positive association in men. Diacyl-PC C40:6 and acyl-alkyl-PC C32:2 were associated with both phenotypes in women.

### Benjamini-Hochberg corrected associations

**Fig 2:**
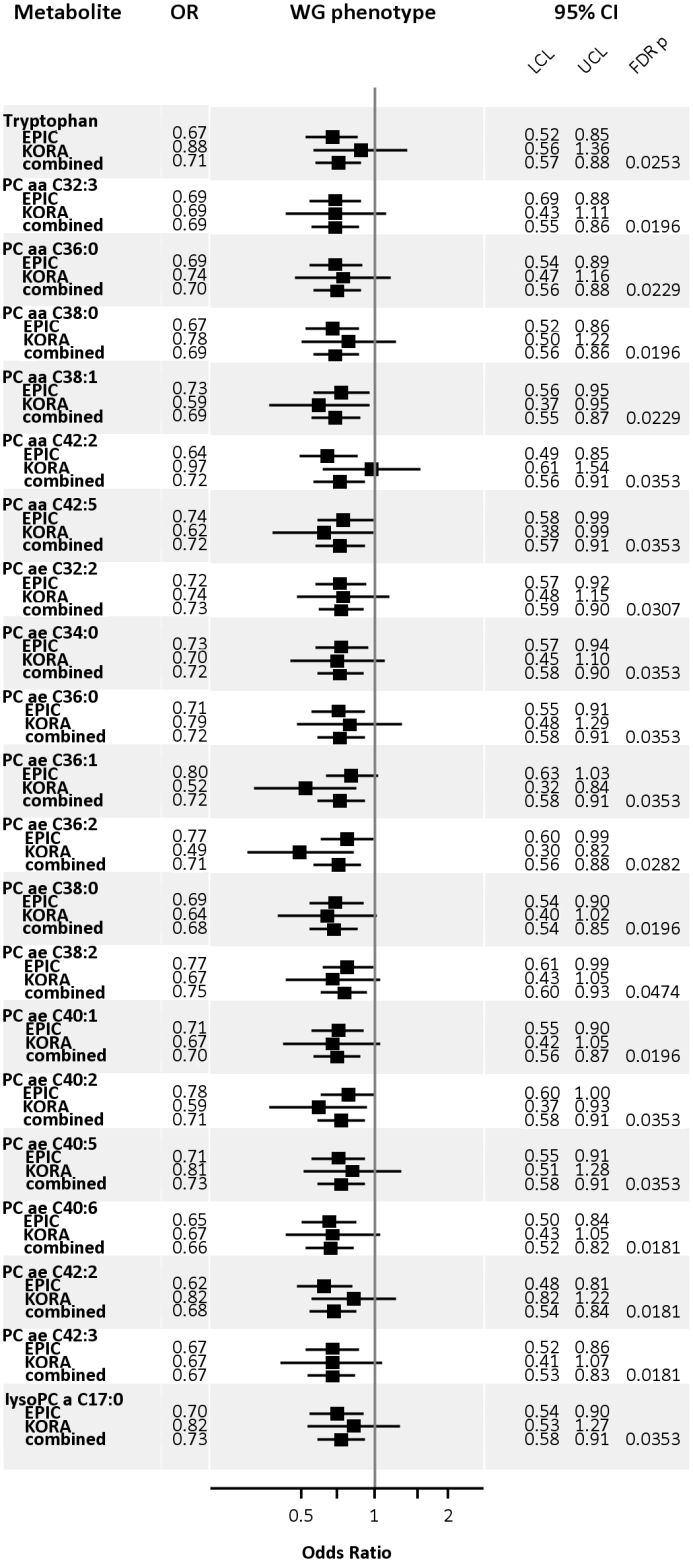
Metabolites showing significant associations with WG phenotype in women after correction for multiple testing. EPIC, European Prospective Investigation into Cancer and Nutrition Potsdam Study; KORA, Cooperative Health Research in the Region of Augsburg Study; PC, phosphatidylcholines; a, acyl; e, alkyl; SM, sphingomyelin.

When we applied the Benjamini-Hochberg method to correct for multiple testing, we identified AA tryptophan, diacyl-PCs C32:3, C36:0, C38:0, C38:1, C42:2, C42:5, acyl-alkyl-PCs C32:2, C34:0, C36:0, C36:1, C36:2, C38:0, C38:2, C40:1, C40:2, C40:5, C40:6, C42:2, C42:3 and lyso-PC C17:0 to be predictive for WG in women (Fig 2). With increasing concentration, each of these metabolites showed inverse associations. All significant associations are within a range of OR 0.66–0.73 (p_FDR_ 0.0181–0.0474), i.e. if the metabolite concentration of selected metabolites increases per one SD the chance of having the WG decreases between 27% and 34%. The lowest odds ratios were observed for acyl-alkyl-PCs C40:6 (OR: 0.66, 95%-CI: 0.52–0.82, p_FDR_ = 0.0181) and C42:3 (OR: 0.67, 95%-CI: 0.53–0.83, p_FDR_ = 0.0181). When comparing results of significant metabolites in women for WG and HG, 17 showed contrasting associations, 4 metabolites had the same direction but much weaker strength of association (Trp, diacyl-PC C42:5, acyl-alkyl-PC C40:1 and C42:2). Complete lists of associations linking both phenotypes with baseline metabolite concentrations are reported in S3–S6 Tables. After multiple testing corrections, associations with WG in women remained significant. In addition, of all the PCs—including those without significant association with WG—the majority showed suggestive negative associations.

None of the metabolites was statistically significantly associated with HG in women or with any of the phenotypes in men.

### Sensitivity and subgroup analysis

Sensitivity analyses were performed for populations without prevalent or incident diseases (EPIC, KORA), a fasting population and in pre-menopausal women (EPIC). Observed associations were robust and independent of diseases, fasting or menopausal status (data not shown). Subgroup analyses for physical activity, age, abdominal obesity and alcohol consumption showed consistent significant results concerning strength and direction of associations in the majority of the examined subgroups compared to the overall population (data not shown). All other results were not significant, whereas the majority of remaining results were also consistent.

In a sensitivity analysis, we addressed potential bias due to drop-out by weighting for drop-out using inverse probability weights for reparticipation at follow-up. Weights were applied in EPIC with a high reparticipation rate of 92% and in DEGS with a comparably low reparticipation rate of 47% [30]. Waist-hip difference as the key variable was robust and did not change noticeably in either of the studies. Therefore no weighting for drop-out was applied.

## Discussion

For Germany, we observed that weight gain is accompanied by a higher gain in waist than in hip circumference. We could identify metabolites whose serum concentrations were associated with the WG and HG phenotypes. Associations with p<0.05 were revealed more often for the WG than for the HG and a particular class of metabolites, the PCs, seems to restrict waist gain, at least in women. In men and for the HG phenotype, no significant association between serum metabolite concentrations and subsequent gain in circumferences remained when taking multiple testing into account.

In our study, comprising two separate cohorts with metabolites measurements the majority of PCs showed suggestive inverse associations with the WG in both sexes. The *de novo* synthesis of PCs is based on two major pathways [45], of which one was reported to be induced by oestrogen [46], and altered sex hormones are involved in the development of abdominal adipose tissue in both men and women [11, 47, 48]. In EPIC, oestrogen and testosterone were not correlated with the majority of PCs (data not shown) in both sexes.

In general, PCs are decreased in obese individuals [49, 50]. Accordingly, PCs were inversely correlated with initial BMI and waist circumference in our study (data not shown). However, due to our prospective study design and statistical modelling we are convinced that this cross-sectional relationship does not interfere with our observation that higher PC levels are inversely associated with WG.

Further research in the EPIC-Potsdam study revealed that waist circumference is only moderately associated with visceral fat mass [51]. At present we cannot rule out that lower gain in waist circumference is particularly related to lower gain in visceral fat mass, which current discussions suggest is involved in the development of chronic diseases like diabetes, hypertension and CVD [52, 53]. Floegel *et al*. [38], for example, reported four of our associated PCs (acyl-alkyl-PCs C40:5, C40:6, C42:3, lyso-PC C17:0) to be associated with a decreased risk of the development of type 2 diabetes. In line with that, insulin-sensitive overweight individuals have higher levels of PCs compared to insulin-resistant overweight individuals [50]. Thus, the deposition of body fat in the abdominal area accompanied by an increase in visceral fat may be an intermediate step linking our WG-associated metabolites to increased risk for the development for type 2 diabetes.

Lyso-PCs are products of hydrolysis from either diacyl- and acyl-alkyl-PCs and associated with body mass and BMI [54]. Metabolic disorders are associated with a reduction in lyso-PC levels [55, 56]. Obese individuals and individuals with impaired glucose tolerance were reported to have decreased plasma levels. This is consistent with our results, where lyso-PC C17:0 was significantly associated with the WG in both sexes and remained significant in women when taking multiple testing into account. Additionally lyso-PCs showed for example, suggestive inverse associations in both sexes with both endpoints.

In summary, higher levels of PCs were reported to be associated with beneficial health [38, 49, 50, 54]. Thus, negative associations may be explained through restricted weight gain due to a more health-conscious lifestyle.

Tryptophan was inversely associated with the WG. Coskun *et al*. [57] reported lower food intake and weight loss in mice treated with tryptophan. As an element of the serotonin metabolism, this AA may be involved in appetite regulation. In our study only weight-gaining participants were included. Subjects with the WG showed significantly higher rates of average annual weight change and lower levels of tryptophan. Therefore higher levels of this AA may have a small effect on caloric restriction and might therefore be protective against waist gain by restricting weight gain.

We were not able to identify significant metabolic predictors of the HG for either sex. Regarding associations before correction for multiple testing, fewer metabolites were associated with the HG compared to the WG. This may be due to the role of visceral adipose tissue as an endocrine organ [14], which is therefore rather predictive due to its integration in physiological processes. This role might be lacking in the case of hip fat mass.

We expected greater differences in the waist-hip distribution between sexes, with more women having a hip-gaining tendency. We observed a stronger correlation between body weight gain and waist-hip difference in men compared to women. Thus, the expected sex differences in body fat distribution seem to be related to the amount of body weight gain, especially in men, and not on waist-hip difference in general.

### Limitations and strengths

It must be emphasized that associations were only observed for the WG phenotype in women when taking multiple testing into account, but not in men. For men, this may be due to reasons of lower statistical power because our analysis included fewer men than women.

Possible sources of bias are the self-reported measures of body weight, waist and hip circumference for follow-up in EPIC. To reduce possible bias, values were corrected using EPIC-specific equations [34, 35].

We assumed that individual weight gain within the follow-up period appeared on the basis of a comparable metabolic profile, as measured at baseline. We are aware that not all metabolites are equally reliable over time. Floegel *et al*. [58] computed the intra-class correlation coefficient (ICC) for p150 metabolites during a 4-month period in fasting individuals. Two metabolites (PC aa C38:1, PC ae C38:2) showing a significant association with the WG in women had a poor ICC of less than 0.4; all other associated metabolites had fair to excellent ICC, with 16 metabolites having at least good reliability of more than 0.51 ICC [58]. Associations of metabolites with an ICC of less than 0.65 may be attenuated and true associations may be even stronger [59].

Human metabolome is age-dependent [60], and follow-up periods in KORA and EPIC were 7.1 and 8.6 years, respectively. Furthermore, aging effects force body fat distribution changes towards more abdominal fat through hormonal changes in both sexes but especially in women due to menopause and oestrogen decline. Sensitivity and subgroup analyses for age and menopausal status showed, that observed associations were robust and therefore not due to physiological aging effects. However, future studies should investigate age-dependent changes of these metabolites.

A limitation in both studies is that not all participants provided their blood sample in a fasting state; nevertheless about 30% in EPIC and 88% in KORA were fasting. To address this potential bias, we performed a sensitivity-analysis in EPIC, showing that our results were robust and independent of fasting status.

A strength of our study is the longitudinal design, which allows us to investigate predictive metabolites of future weight gain phenotypes in well-described cohort studies.

To our knowledge, we are the first group to use this innovative average annual waist-hip difference to characterize waist- and hip-gaining phenotypes. This difference has the advantage of better interpretability compared to the WHR because of its absolute changes expressed in cm/yr. It is also a novelty to identify metabolites as determinants for those phenotypes using a targeted metabolomics approach.

We were able to replicate and summarize metabolomics-results in two independent well-described German study populations, therefore we can be sure that the observed associations are not study-specific.

## Conclusion

The targeted metabolomics approach revealed metabolites with inverse associations for the WG. With regard to further elucidation of the role of the identified metabolites in terms of primary prevention and common pathophysiological pathways, further studies are needed to investigate in particular phospholipid-metabolism as a determinant of body fat deposition.

## Supporting Information

S1 Table Characteristics of women included in the analysis according to endpoint categories. (DOCX)

S2 Table Characteristics of men included in the analysis according to endpoint categories. (DOCX)

S3 Table Association of metabolites with waist-gaining phenotype in women in the combined fixed-effect meta-analysis and by specific study. (DOCX)

S4 Table Association of metabolites with hip-gaining phenotype in women in the combined fixed-effect meta-analysis and by specific study. (DOCX)

S5 Table Association of metabolites with waist-gaining phenotype in men in the combined fixed-effect meta-analysis and by specific study. (DOCX)

S6 Table Association of metabolites with hip-gaining phenotype in men in the combined fixed-effect meta-analysis and by specific study. (DOCX)
