# Supplementary material for: Specific Metabolic Markers Are Associated with Future Waist-Gaining Phenotype in Women
Source: PLoS One. 2016 Jun 20;11(6):e0157733. doi: 10.1371/journal.pone.0157733 (PMC4920591; doi:10.1371/journal.pone.0157733)
Supplement: S1 Table — (DOCX) [file pone.0157733.s001.docx]

Table S1: Characteristics of men included in the analysis according to endpoint categories ^a^

|  | **reference category** | | | | | | | | |  | **WG phenotype** | | | | | | | | |  | **HG phenotype** | | | | | | | | | |
| --- | --- | --- | --- | --- | --- | --- | --- | --- | --- | --- | --- | --- | --- | --- | --- | --- | --- | --- | --- | --- | --- | --- | --- | --- | --- | --- | --- | --- | --- | --- |
|  | **EPIC  (n=504)** | | | **KORA (n=231)** | | | **DEGS**  **(n=709)** | | |  | **EPIC  (n=63)** | | | **KORA (n=29)** | | | **DEGS**  **(n=88)** | | |  | **EPIC  (n=62)** | | | **KORA (n=29)** | | | | **DEGS**  **(n=87)** | | |
| **baseline characteristics** | **Mean** |  | **SD** | **Mean** |  | **SD** | **Mean** |  | **SD** |  | **Mean** |  | **SD** | **Mean** |  | **SD** | **Mean** |  | **SD** |  | **Mean** |  | **SD** | **Mean** |  | **SD** | | **Mean** |  | **SD** |
| age at recruitment | 51.5 | ± | 7.9 | 62.4 | ± | 5.3 | 41.8 | ± | 12.81 |  | 49.3 | ± | 8.0 | 63.3 | ± | 5.2 | 41.8 | ± | 13.13 |  | 53.8 | ± | 8.3 | 64.0 | ± | | 4.9 | 41.1 | ± | 12.1 |
| body weight (kg) | 80.8 | ± | 11.0 | 83.1 | ± | 10.9 | 82.7 | ± | 11.61 |  | 85.3 | ± | 13.8 | 85.5 | ± | 8.8 | 84.9 | ± | 11.84 |  | 78.8 | ± | 10.6 | 89.9 | ± | | 13.4 | 85.4 | ± | 12.6 |
| waist circumference (cm) | 93.0 | ± | 9.3 | 99.1 | ± | 8.7 | 94.6 | ± | 10.40 |  | 91.2 | ± | 12.1 | 101.5 | ± | 7.4 | 94.7 | ± | 10.99 |  | 94.8 | ± | 9.0 | 106.8 | ± | | 10.6 | 98.2 | ± | 11.8 |
| hip circumference (cm) | 99.6 | ± | 5.8 | 104.3 | ± | 6.4 | 104.6 | ± | 6.02 |  | 102.3 | ± | 7.0 | 105.7 | ± | 5.8 | 106.1 | ± | 5.83 |  | 96.8 | ± | 5.3 | 107.2 | ± | | 7.9 | 105.1 | ± | 8.3 |
| BMI (kg/m²) | 26.5 | ± | 3.4 | 27.8 | ± | 3.3 | 26.42 | ± | 3.39 |  | 26.8 | ± | 3.9 | 29.0 | ± | 2.4 | 27.3 | ± | 3.15 |  | 26.5 | ± | 3.2 | 30.1 | ± | | 4.5 | 27.2 | ± | 4.3 |
| WHR | 0.93 | ± | 0.06 | 0.95 | ± | 0.05 | 0.90 | ± | 0.07 |  | 0.89 | ± | 0.08 | 0.96 | ± | 0.04 | 0.89 | ± | 0.08 |  | 0.98 | ± | 0.05 | 1.00 | ± | | 0.04 | 0.93 | ± | 0.07 |
| Prevalence of abdominal obesity ^b^ | 17.7 | | | 30.7 | | | 23.8 | | |  | 20.6 | | | 41.4 | | | 22.3 | | |  | 22.6 | | | 65.5 | | | | 34.5 | | |
| alcohol consumption (g/d) | 24.5 | ± | 29.2 | 26.4 | ± | 26.7 | 15.1 | ± | 19.01 |  | 23.2 | ± | 19.7 | 21.1 | ± | 21.3 | 18.6 | ± | 23.2 |  | 25.9 | ± | 21.0 | 26.4 | ± | | 30.4 | 15.8 | ± | 18.5 |
|  |  |  |  |  |  |  |  |  |  |  |  |  |  |  |  |  |  |  |  |  |  |  |  |  |  | |  |  |  |  |
| **average changes per year** |  |  |  |  |  |  |  |  |  |  |  |  |  |  |  |  |  |  |  |  |  |  |  |  |  | |  |  |  |  |
| weight (%/yr) | 0.59 | ± | 0.45 | 0.59 | ± | 0.49 | 0.63 | ± | 0.57 |  | 1.10 | ± | 0.70 | 1.15 | ± | 0.96 | 1.11 | ± | 0.83 |  | 0.41 | ± | 0.40 | 0.49 | ± | | 0.43 | 0.50 | ± | 0.39 |
| waist circumference (%/yr) | 1.29 | ± | 0.58 | 0.74 | ± | 0.50 | 0.60 | ± | 0.54 |  | 2.55 | ± | 0.89 | 1.61 | ± | 0.71 | 1.53 | ± | 0.86 |  | 0.49 | ± | 0.53 | 0.22 | ± | | 0.49 | -0.08 | ± | 0.42 |
| hip circumference (%/yr) | 0.47 | ± | 0.38 | 0.38 | ± | 0.44 | -0.11 | ± | 0.32 |  | 0.43 | ± | 0.50 | 0.29 | ± | 0.51 | -0.21 | ± | 0.47 |  | 0.78 | ± | 0.56 | 0.94 | ± | | 0.50 | 0.15 | ± | 0.44 |
|  |  |  |  |  |  |  |  |  |  |  |  |  |  |  |  |  |  |  |  |  |  |  |  |  |  | |  |  |  |  |
| **smoking status** |  |  |  |  |  |  |  |  |  |  |  |  |  |  |  |  |  |  |  |  |  |  |  |  |  | |  |  |  |  |
| never smoker |  |  | 30.4 |  |  | 32.5 |  |  | 38.1 |  |  |  | 22.2 |  |  | 24.1 |  |  | 37.3 |  |  |  | 22.6 |  |  | | 31.0 |  |  | 45.0 |
| former smoker |  |  | 44.0 |  |  | 50.6 |  |  | 26.2 |  |  |  | 41.3 |  |  | 48.3 |  |  | 24.3 |  |  |  | 62.9 |  |  | | 55.2 |  |  | 31.8 |
| current smoker |  |  | 25.6 |  |  | 16.9 |  |  | 35.7 |  |  |  | 36.5 |  |  | 27.6 |  |  | 38.4 |  |  |  | 12.5 |  |  | | 13.8 |  |  | 23.2 |
|  |  |  |  |  |  |  |  |  |  |  |  |  |  |  |  |  |  |  |  |  |  |  |  |  |  | |  |  |  |  |
| **physical activity** |  |  |  |  |  |  |  |  |  |  |  |  |  |  |  |  |  |  |  |  |  |  |  |  |  | |  |  |  |  |
| < 1 h/week |  |  | 64.9 |  |  | 58 |  |  | 56.4 |  |  |  | 60.3 |  |  | 55.2 |  |  | 62.0 |  |  |  | 69.3 |  |  | | 69.0 |  |  | 54.0 |
| >1-2h/week |  |  | 18.1 |  |  | 22.1 |  |  | 17.6 |  |  |  | 19.1 |  |  | 27.6 |  |  | 11.0 |  |  |  | 11.3 |  |  | | 24.1 |  |  | 17.0 |
| > 2h/week |  |  | 17.0 |  |  | 19.9 |  |  | 26.0 |  |  |  | 20.6 |  |  | 17.2 |  |  | 27.0 |  |  |  | 19.4 |  |  | | 6.9 |  |  | 29.0 |
|  |  |  |  |  |  |  |  |  |  |  |  |  |  |  |  |  |  |  |  |  |  |  |  |  |  | |  |  |  |  |
| **prevalent diseases** |  |  | 19.3 |  |  | 30.3 |  |  | 7.1 |  |  |  | 22.2 |  |  | 37.9 |  |  | 14.1 |  |  |  | 21.0 |  |  | | 37.9 |  |  | 7.4 |

BMI, body mass index; WHR, waist-to-hip ratio.

^a^Values are mean ± SD or percent (%);

^b^DEGS is standardized to the structure of the German population at 31.12.1997;

^c^abdominal obesity defined as waist circumference >88 cm (women) / >102 cm (men)
