# Supplementary material for: Specific Metabolic Markers Are Associated with Future Waist-Gaining Phenotype in Women
Source: PLoS One. 2016 Jun 20;11(6):e0157733. doi: 10.1371/journal.pone.0157733 (PMC4920591; doi:10.1371/journal.pone.0157733)
Supplement: S2 Table — (DOCX) [file pone.0157733.s002.docx]

Table S2: Characteristics of women included in the analysis according to endpoint categories ^a^

|  |  | **reference category** | | | | | | | | |  | **WG phenotype** | | | | | | | | |  | **HG phenotype** | | | | | | | | | | | | | | | | |
| --- | --- | --- | --- | --- | --- | --- | --- | --- | --- | --- | --- | --- | --- | --- | --- | --- | --- | --- | --- | --- | --- | --- | --- | --- | --- | --- | --- | --- | --- | --- | --- | --- | --- | --- | --- | --- | --- | --- |
|  |  | **EPIC  (n=813)** | | | **KORA (n=241)** | | | **DEGS**  **(n=806)** | | |  | **EPIC  (n=101)** | | | **KORA (n=31)** | | | **DEGS**  **(n=100)** | | |  | **EPIC  (n=101)** | | | | | | | **KORA (n=31)** | | | | | **DEGS**  **(n=100)** | | | | |
| **baseline characteristics** |  | **Mean** |  | **SD** | **Mean** |  | **SD** | **Mean** |  | **SD** |  | **Mean** |  | **SD** | **Mean** |  | **SD** | **Mean** |  | **SD** |  | **Mean** | | |  | | **SD** | | **Mean** | |  | **SD** | | **Mean** | |  | **SD** | |
| age at recruitment |  | 47.8 | ± | 8.86 | 62.8 | ± | 5.2 | 43.7 | ± | 11.8 |  | 9.0 | ± | 9.5 | 61.5 | ± | 4.8 | 46.45 | ± | 11.50 |  | 50.5 | | | ± | | 9.2 | | 64.4 | | ± | 4.9 | | 37.88 | | ± | 10.90 | |
| body weight (kg) |  | 66.0 | ± | 11.4 | 70.9 | ± | 11.5 | 67.8 | ± | 11.8 |  | 64.9 | ± | 10.1 | 75.4 | ± | 14.8 | 78.51 | ± | 17.50 |  | 76.7 | | | ± | | 16.4 | | 76.6 | | ± | 11.3 | | 62.90 | | ± | 11.38 | |
| waist circumference (cm) |  | 78.4 | ± | 10.17 | 88.5 | ± | 10.5 | 81.6 | ± | 10.6 |  | 74.2 | ± | 8.37 | 87.4 | ± | 11.1 | 90.04 | ± | 12.97 |  | 92.2 | | | ± | | 14.2 | | 95.8 | | ± | 11.8 | | 78.04 | | ± | 13.10 | |
| hip circumference (cm) |  | 99.9 | ± | 8.29 | 106.0 | ± | 8.5 | 103.6 | ± | 8.8 |  | 100.8 | ± | 7.6 | 110.2 | ± | 11.5 | 113.47 | ± | 14.47 |  | 104.6 | | | ± | | 11.0 | | 111.0 | | ± | 9.0 | | 99.04 | | ± | 9.30 | |
| BMI (kg/m²) |  | 24.8 | ± | 4.08 | 28.0 | ± | 4.3 | 25.2 | ± | 4.1 |  | 24.36 | ± | 3.71 | 29.1 | ± | 6.3 | 29.78 | ± | 6.79 |  | 28.6 | | | ± | | 5.6 | | 30.4 | | ± | 4.9 | | 23.47 | | ± | 4.51 | |
| WHR |  | 0.78 | ± | 0.06 | 0.83 | ± | 0.06 | 0.79 | ± | 0.06 |  | 0.74 | ± | 0.05 | 0.79 | ± | 0.05 | 0.79 | ± | 0.05 |  | 0.88 | | | ± | | 0.07 | | 0.86 | | ± | 0.06 | | 0.79 | | ± | 0.08 | |
| abdominal obesity ^b^ |  | 17.0 | | | 49.4 | | | 26.9 | | |  | 9.9 | | | 45.2 | | | 51.5 | | |  | | 57.4 | | | | | 74.2 | | | | | | 17.0 | | | | |
| alcohol consumption (g/d) |  | 8.6 | ± | 10.22 | 7.5 | ± | 11.2 | 4.9 | ± | 8.6 |  | 8.9 | ± | 12.9 | 6.1 | ± | 10.0 | 4.53 | ± | 9.01 |  | 8.5 | | ± | | 10.6 | | 4.0 | | ± | | | 5.8 | 3.20 | ± | | | 5.61 |
|  |  |  |  |  |  |  |  |  |  |  |  |  |  |  |  |  |  |  |  |  |  |  | |  | |  | |  | |  | | |  |  |  | | |  |
| **Averaged percentage changes per year** |  |  |  |  |  |  |  |  |  |  |  |  |  |  |  |  |  |  |  |  |  |  | |  | |  | |  | |  | | |  |  |  | | |  |
| weight (%/yr) |  | 0.94 | ± | 0.80 | 0.72 | ± | 0.69 | 0.75 | ± | 0.59 |  | 1.21 | ± | 0.85 | 1.08 | ± | 1.16 | 0.95 | ± | 0.76 |  | 0.77 | | ± | | 0.71 | | 0.95 | | ± | | | 0.95 | 0.80 | ± | | | 0.60 |
| waist circumference (%/yr) |  | 1.48 | ± | 0.72 | 0.94 | ± | 0.62 | 0.93 | ± | 0.68 |  | 2.56 | ± | 1.03 | 2.07 | ± | 0.97 | 1.84 | ± | 0.81 |  | 0.48 | | ± | | 0.78 | | 0.14 | | ± | | | 0.85 | 0.27 | ± | | | 0.76 |
| hip circumference (%/yr) |  | 0.54 | ± | 0.48 | 0.51 | ± | 0.49 | 0.15 | ± | 0.38 |  | 0.27 | ± | 0.58 | 0.34 | ± | 0.59 | -0.13 | ± | 0.50 |  | 0.81 | | ± | | 0.62 | | 0.93 | | ± | | | 0.72 | 0.65 | ± | | | 0.44 |
|  |  |  |  |  |  |  |  |  |  |  |  |  |  |  |  |  |  |  |  |  |  |  | |  | |  | |  | |  | | |  |  |  | | |  |
| **smoking status** |  |  |  |  |  |  |  |  |  |  |  |  |  |  |  |  |  |  |  |  |  |  | |  | |  | |  | |  | | |  |  |  | | |  |
| never smoker |  |  |  | 58.3 |  |  | 71.4 |  |  | 53.9 |  |  |  | 62.4 |  |  | 64.5 |  |  | 60.5 |  |  | |  | | 60.4 | |  | |  | | | 71.0 |  |  | | | 59.4 |
| former smoker |  |  |  | 23.4 |  |  | 21.2 |  |  | 17.0 |  |  |  | 21.8 |  |  | 29.0 |  |  | 13.7 |  |  | |  | | 24.8 | |  | |  | | | 22.6 |  |  | | | 15.5 |
| current smoker |  |  |  | 18.3 |  |  | 7.5 |  |  | 29.1 |  |  |  | 15.8 |  |  | 6.5 |  |  | 25.8 |  |  | |  | | 14.8 | |  | |  | | | 6.4 |  |  | | | 25.1 |
|  |  |  |  |  |  |  |  |  |  |  |  |  |  |  |  |  |  |  |  |  |  |  | |  | |  | |  | |  | | |  |  |  | | |  |
| **menopause** |  |  |  | 21.7 |  |  | 95.4 |  |  | 60.2 |  |  |  | 25.7 |  |  | 87.1 |  |  | 44.6 |  |  | |  | | 28.7 | |  | |  | | | 96.8 |  |  | | | 75.8 |
|  |  |  |  |  |  |  |  |  |  |  |  |  |  |  |  |  |  |  |  |  |  |  | |  | |  | |  | |  | | |  |  |  | | |  |
| **physical activity** |  |  |  |  |  |  |  |  |  |  |  |  |  |  |  |  |  |  |  |  |  |  | |  | |  | |  | |  | | |  |  |  | | |  |
| < 1 h/week* |  |  |  | 60.0 |  |  | 49.8 |  |  | 41.4 |  |  |  | 58.4 |  |  | 58.1 |  |  | 65.6 |  |  | |  | | 62.4 | |  | |  | | | 61.3 |  |  | | | 53.5 |
| 1-2h/week |  |  |  | 25.6 |  |  | 31.1 |  |  | 21.1 |  |  |  | 24.8 |  |  | 29.0 |  |  | 19.7 |  |  | |  | | 29.7 | |  | |  | | | 32.2 |  |  | | | 19.5 |
| > 2h/week |  |  |  | 14.4 |  |  | 19.1 |  |  | 20.2 |  |  |  | 16.8 |  |  | 12.9 |  |  | 14.8 |  |  | |  | | 7.9 | |  | |  | | | 6.5 |  |  | | | 23.9 |
|  |  |  |  |  |  |  |  |  |  |  |  |  |  |  |  |  |  |  |  |  |  |  | |  | |  | |  | |  | | |  |  |  | | |  |
| **prevalent diseases** |  |  |  | 14.8 |  |  | 23.7 |  |  | 7.3 |  |  |  | 10.9 |  |  | 12.9 |  |  | 12.2 |  |  | |  | | 19.8 | |  | |  | | | 19.3 |  |  | | | 5.5 |

BMI, body mass index; WHR, waist-to-hip ratio.

^a^Values are mean ± SD or percent (%);

^b^DEGS is standardized to the structure of the German population at 31.12.1997;

^c^abdominal obesity defined as waist circumference >88 cm (women) / >102 cm (men)
