# Supplementary material for: Specific Metabolic Markers Are Associated with Future Waist-Gaining Phenotype in Women
Source: PLoS One. 2016 Jun 20;11(6):e0157733. doi: 10.1371/journal.pone.0157733 (PMC4920591; doi:10.1371/journal.pone.0157733)
Supplement: S3 Table — (DOCX) [file pone.0157733.s003.docx]

Table S3 Association of metabolites with waist-gaining phenotype in women in the combined fixed-effect meta-analysis and by specific study

|  | **overall** | | | |  | **EPIC-Potsdam** | | | | |  | **KORA** | | | | |
| --- | --- | --- | --- | --- | --- | --- | --- | --- | --- | --- | --- | --- | --- | --- | --- | --- |
| **Amino Acids** | **OR** | **LCL** | **UCL** | **FDR p** |  | **OR** | **LCL** | **UCL** | **uncorrected  p value** | **meta weight** |  | **OR** | **LCL** | **UCL** | **uncorrected  p value** | **meta weight** |
| Arg | 0.81 | 0.66 | 1.00 | 0.1428 |  | 0.81 | 0.64 | 1.04 | 0.0949 | 75.3% |  | 0.80 | 0.53 | 1.22 | 0.3026 | 24.7% |
| Gln | 0.87 | 0.71 | 1.07 | 0.3315 |  | 0.81 | 0.64 | 1.02 | 0.0686 | 76.5% |  | 1.11 | 0.73 | 1.70 | 0.6169 | 23.5% |
| Gly | 0.89 | 0.73 | 1.09 | 0.3993 |  | 0.78 | 0.61 | 1.00 | 0.0453 | 66.6% |  | 1.17 | 0.83 | 1.66 | 0.3650 | 33.4% |
| His | 0.94 | 0.77 | 1.15 | 0.6657 |  | 0.92 | 0.72 | 1.16 | 0.4815 | 73.8% |  | 1.00 | 0.67 | 1.50 | 0.9834 | 26.2% |
| Met | 0.81 | 0.64 | 1.03 | 0.2272 |  | 0.70 | 0.52 | 0.93 | 0.0143 | 69.4% |  | 1.14 | 0.74 | 1.77 | 0.5544 | 30.6% |
| Orn | 0.94 | 0.76 | 1.14 | 0.6395 |  | 0.88 | 0.69 | 1.11 | 0.2773 | 71.7% |  | 1.11 | 0.76 | 1.62 | 0.6060 | 28.3% |
| Phe | 0.82 | 0.66 | 1.02 | 0.2013 |  | 0.82 | 0.64 | 1.05 | 0.1141 | 75.8% |  | 0.83 | 0.53 | 1.29 | 0.4084 | 24.2% |
| Pro | 1.00 | 0.81 | 1.23 | 0.9995 |  | 1.00 | 0.80 | 1.26 | 0.9943 | 82.6% |  | 0.99 | 0.60 | 1.62 | 0.9693 | 17.4% |
| Ser | 0.88 | 0.71 | 1.08 | 0.3489 |  | 0.75 | 0.59 | 0.97 | 0.0279 | 68.6% |  | 1.22 | 0.84 | 1.77 | 0.3053 | 31.4% |
| Thr | 0.95 | 0.76 | 1.18 | 0.7224 |  | 0.83 | 0.65 | 1.07 | 0.1498 | 76.5% |  | 1.43 | 0.92 | 2.24 | 0.1150 | 23.5% |
| Trp | 0.71 | 0.57 | 0.88 | 0.0253 |  | 0.67 | 0.52 | 0.85 | 0.0013 | 75.8% |  | 0.88 | 0.56 | 1.36 | 0.5580 | 24.2% |
| Tyr | 0.93 | 0.75 | 1.15 | 0.6229 |  | 0.88 | 0.69 | 1.13 | 0.3076 | 75.3% |  | 1.10 | 0.71 | 1.69 | 0.6725 | 24.7% |
| Val | 0.91 | 0.71 | 1.15 | 0.5541 |  | 0.83 | 0.62 | 1.10 | 0.1947 | 69.7% |  | 1.12 | 0.73 | 1.74 | 0.5991 | 30.3% |
| **Hexose** |  |  |  |  |  |  |  |  |  |  |  |  |  |  |  |  |
| H1 | 1.00 | 0.79 | 1.26 | 0.9995 |  | 0.92 | 0.70 | 1.21 | 0.5631 | 70.4% |  | 1.21 | 0.79 | 1.85 | 0.3759 | 29.6% |
| **Acylcarnitines** |  |  |  |  |  |  |  |  |  |  |  |  |  |  |  |  |
| C0 | 1.02 | 0.83 | 1.25 | 0.9018 |  | 0.97 | 0.77 | 1.22 | 0.7931 | 75.8% |  | 1.20 | 0.80 | 1.81 | 0.3826 | 24.2% |
| C2 | 0.92 | 0.75 | 1.13 | 0.5541 |  | 0.89 | 0.71 | 1.12 | 0.3196 | 80.6% |  | 1.05 | 0.66 | 1.67 | 0.8461 | 19.4% |
| C3 | 1.06 | 0.87 | 1.30 | 0.6657 |  | 1.01 | 0.80 | 1.28 | 0.9401 | 74.9% |  | 1.25 | 0.83 | 1.87 | 0.2852 | 25.1% |
| C5-OH (C3-DC-M) | 1.14 | 0.94 | 1.38 | 0.3315 |  | 1.16 | 0.94 | 1.43 | 0.1724 | 80.0% |  | 1.07 | 0.70 | 1.64 | 0.7450 | 20.0% |
| C7-DC | 0.88 | 0.71 | 1.09 | 0.3853 |  | 0.92 | 0.73 | 1.16 | 0.4776 | 86.7% |  | 0.66 | 0.37 | 1.19 | 0.1714 | 13.3% |
| C9 | 0.90 | 0.73 | 1.12 | 0.4898 |  | 0.94 | 0.75 | 1.19 | 0.6249 | 83.4% |  | 0.72 | 0.43 | 1.21 | 0.2148 | 16.6% |
| C10 | 0.81 | 0.63 | 1.05 | 0.2755 |  | 0.85 | 0.64 | 1.13 | 0.2646 | 82.4% |  | 0.65 | 0.35 | 1.21 | 0.1749 | 17.6% |
| C10:2 | 1.04 | 0.85 | 1.27 | 0.7788 |  | 1.07 | 0.86 | 1.34 | 0.5337 | 78.3% |  | 0.93 | 0.61 | 1.42 | 0.7330 | 21.7% |
| C14:1 | 0.86 | 0.69 | 1.07 | 0.3315 |  | 0.88 | 0.70 | 1.12 | 0.2972 | 81.5% |  | 0.77 | 0.47 | 1.28 | 0.3165 | 18.5% |
| C14:2 | 0.93 | 0.76 | 1.14 | 0.6039 |  | 0.92 | 0.73 | 1.15 | 0.4503 | 79.2% |  | 0.98 | 0.63 | 1.52 | 0.9386 | 20.8% |
| C16 | 0.77 | 0.62 | 0.96 | 0.0815 |  | 0.82 | 0.64 | 1.04 | 0.1041 | 79.4% |  | 0.63 | 0.39 | 1.02 | 0.0613 | 20.6% |
| C18 | 0.79 | 0.63 | 0.98 | 0.1102 |  | 0.82 | 0.64 | 1.05 | 0.1175 | 79.7% |  | 0.67 | 0.41 | 1.10 | 0.1137 | 20.3% |
| C18:1 | 0.85 | 0.69 | 1.05 | 0.2965 |  | 0.86 | 0.68 | 1.09 | 0.2191 | 79.9% |  | 0.81 | 0.50 | 1.31 | 0.3902 | 20.1% |
| C18:2 | 0.98 | 0.80 | 1.19 | 0.8721 |  | 0.95 | 0.76 | 1.19 | 0.6415 | 81.2% |  | 1.10 | 0.69 | 1.76 | 0.6883 | 18.8% |
| **diacyl-Phosphatidylcholines** | | |  |  |  |  |  |  |  |  |  |  |  |  |  |  |
| PC aa C28:1 | 0.79 | 0.63 | 0.98 | 0.1037 |  | 0.91 | 0.72 | 1.15 | 0.4268 | 83.0% |  | 0.39 | 0.23 | 0.67 | 0.0005 | 17.0% |
| PC aa C30:0 | 0.88 | 0.71 | 1.08 | 0.3488 |  | 0.92 | 0.73 | 1.17 | 0.5041 | 77.6% |  | 0.73 | 0.47 | 1.13 | 0.1563 | 22.4% |
| PC aa C32:0 | 0.84 | 0.68 | 1.05 | 0.2926 |  | 0.82 | 0.64 | 1.05 | 0.1184 | 77.1% |  | 0.92 | 0.58 | 1.46 | 0.7313 | 22.9% |
| PC aa C32:1 | 0.90 | 0.72 | 1.12 | 0.4773 |  | 0.89 | 0.69 | 1.14 | 0.3598 | 78.1% |  | 0.91 | 0.57 | 1.47 | 0.7124 | 21.9% |
| PC aa C32:2 | 0.85 | 0.69 | 1.06 | 0.2995 |  | 0.93 | 0.73 | 1.18 | 0.5345 | 79.5% |  | 0.62 | 0.39 | 0.99 | 0.0456 | 20.5% |
| PC aa C32:3 | 0.69 | 0.55 | 0.86 | 0.0196 |  | 0.69 | 0.54 | 0.88 | 0.0034 | 78.4% |  | 0.69 | 0.43 | 1.11 | 0.1297 | 21.6% |
| PC aa C34:1 | 0.88 | 0.71 | 1.09 | 0.3901 |  | 0.93 | 0.73 | 1.17 | 0.5253 | 80.7% |  | 0.73 | 0.45 | 1.18 | 0.1974 | 19.3% |
| PC aa C34:2 | 0.88 | 0.71 | 1.08 | 0.3594 |  | 0.94 | 0.75 | 1.18 | 0.5856 | 81.5% |  | 0.66 | 0.41 | 1.07 | 0.0916 | 18.5% |
| PC aa C34:3 | 0.85 | 0.69 | 1.05 | 0.2965 |  | 0.85 | 0.67 | 1.08 | 0.1813 | 76.6% |  | 0.87 | 0.56 | 1.33 | 0.5173 | 23.4% |
| PC aa C34:4 | 0.96 | 0.78 | 1.18 | 0.7788 |  | 1.05 | 0.83 | 1.31 | 0.6993 | 80.2% |  | 0.68 | 0.43 | 1.07 | 0.0947 | 19.8% |
| PC aa C36:0 | 0.70 | 0.56 | 0.88 | 0.0229 |  | 0.69 | 0.54 | 0.89 | 0.0043 | 76.5% |  | 0.74 | 0.47 | 1.16 | 0.1894 | 23.5% |
| PC aa C36:1 | 0.87 | 0.71 | 1.07 | 0.3409 |  | 0.90 | 0.71 | 1.15 | 0.3995 | 75.9% |  | 0.78 | 0.51 | 1.19 | 0.2417 | 24.1% |
| PC aa C36:2 | 0.87 | 0.71 | 1.07 | 0.3315 |  | 0.92 | 0.73 | 1.16 | 0.4729 | 79.1% |  | 0.70 | 0.45 | 1.09 | 0.1174 | 20.9% |
| PC aa C36:3 | 0.91 | 0.74 | 1.12 | 0.5111 |  | 1.00 | 0.80 | 1.26 | 0.9989 | 83.2% |  | 0.56 | 0.34 | 0.94 | 0.0279 | 16.8% |
| PC aa C36:4 | 1.03 | 0.84 | 1.27 | 0.8400 |  | 1.12 | 0.90 | 1.40 | 0.3177 | 84.6% |  | 0.66 | 0.39 | 1.11 | 0.1172 | 15.4% |
| PC aa C36:5 | 0.85 | 0.66 | 1.08 | 0.3315 |  | 0.84 | 0.64 | 1.12 | 0.2315 | 76.1% |  | 0.86 | 0.52 | 1.41 | 0.5475 | 23.9% |
| PC aa C36:6 | 0.77 | 0.61 | 0.97 | 0.0961 |  | 0.81 | 0.62 | 1.06 | 0.1206 | 76.0% |  | 0.65 | 0.41 | 1.05 | 0.0767 | 24.0% |
| PC aa C38:0 | 0.69 | 0.56 | 0.86 | 0.0196 |  | 0.67 | 0.52 | 0.86 | 0.0016 | 76.1% |  | 0.78 | 0.50 | 1.22 | 0.2796 | 23.9% |
| PC aa C38:1 | 0.69 | 0.55 | 0.87 | 0.0229 |  | 0.73 | 0.56 | 0.95 | 0.0173 | 76.0% |  | 0.59 | 0.37 | 0.95 | 0.0281 | 24.0% |
| PC aa C38:3 | 0.94 | 0.75 | 1.18 | 0.7030 |  | 1.09 | 0.85 | 1.39 | 0.5081 | 82.4% |  | 0.48 | 0.28 | 0.82 | 0.0068 | 17.6% |
| PC aa C38:4 | 1.00 | 0.81 | 1.23 | 0.9995 |  | 1.10 | 0.87 | 1.38 | 0.4191 | 81.6% |  | 0.66 | 0.41 | 1.07 | 0.0900 | 18.4% |
| PC aa C38:5 | 0.85 | 0.68 | 1.05 | 0.2965 |  | 0.88 | 0.69 | 1.13 | 0.3184 | 78.5% |  | 0.74 | 0.46 | 1.18 | 0.2025 | 21.5% |
| PC aa C38:6 | 0.76 | 0.61 | 0.94 | 0.0572 |  | 0.81 | 0.63 | 1.04 | 0.0986 | 77.0% |  | 0.60 | 0.38 | 0.94 | 0.0259 | 23.0% |
| PC aa C40:2 | 0.75 | 0.59 | 0.97 | 0.0996 |  | 0.71 | 0.52 | 0.97 | 0.0321 | 66.1% |  | 0.85 | 0.55 | 1.30 | 0.4479 | 33.9% |
| PC aa C40:3 | 0.76 | 0.60 | 0.95 | 0.0789 |  | 0.75 | 0.58 | 0.99 | 0.0389 | 72.8% |  | 0.77 | 0.50 | 1.19 | 0.2393 | 27.2% |
| PC aa C40:4 | 0.98 | 0.79 | 1.21 | 0.9018 |  | 1.05 | 0.83 | 1.33 | 0.6710 | 82.2% |  | 0.70 | 0.43 | 1.17 | 0.1749 | 17.8% |
| PC aa C40:5 | 0.85 | 0.68 | 1.07 | 0.3212 |  | 0.91 | 0.71 | 1.18 | 0.4835 | 77.5% |  | 0.67 | 0.42 | 1.07 | 0.0960 | 22.5% |
| PC aa C40:6 | 0.74 | 0.59 | 0.93 | 0.0535 |  | 0.78 | 0.60 | 1.02 | 0.0669 | 74.3% |  | 0.63 | 0.40 | 0.99 | 0.0465 | 25.7% |
| PC aa C42:0 | 0.79 | 0.64 | 0.98 | 0.1102 |  | 0.72 | 0.56 | 0.92 | 0.0097 | 73.4% |  | 1.05 | 0.69 | 1.59 | 0.8310 | 26.6% |
| PC aa C42:1 | 0.77 | 0.62 | 0.96 | 0.0798 |  | 0.71 | 0.55 | 0.91 | 0.0070 | 73.5% |  | 0.99 | 0.65 | 1.51 | 0.9569 | 26.5% |
| PC aa C42:2 | 0.72 | 0.56 | 0.91 | 0.0353 |  | 0.64 | 0.49 | 0.85 | 0.0017 | 73.6% |  | 0.97 | 0.61 | 1.54 | 0.9040 | 26.4% |
| PC aa C42:4 | 0.85 | 0.69 | 1.04 | 0.2646 |  | 0.84 | 0.67 | 1.06 | 0.1455 | 75.3% |  | 0.86 | 0.57 | 1.30 | 0.4728 | 24.7% |
| PC aa C42:5 | 0.72 | 0.57 | 0.91 | 0.0353 |  | 0.75 | 0.58 | 0.99 | 0.0393 | 76.1% |  | 0.62 | 0.38 | 0.99 | 0.0464 | 23.9% |
| PC aa C42:6 | 0.76 | 0.61 | 0.95 | 0.0785 |  | 0.78 | 0.60 | 1.00 | 0.0540 | 76.6% |  | 0.71 | 0.45 | 1.13 | 0.1474 | 23.4% |
| **acyl-alkyl-Phosphatidylcholines** | | |  |  |  |  |  |  |  |  |  |  |  |  |  |  |
| PC ae C30:0 | 0.76 | 0.61 | 0.94 | 0.0572 |  | 0.79 | 0.62 | 1.01 | 0.0560 | 78.2% |  | 0.66 | 0.41 | 1.04 | 0.0723 | 21.8% |
| PC ae C30:2 | 0.74 | 0.59 | 0.94 | 0.0572 |  | 0.82 | 0.64 | 1.06 | 0.1306 | 83.2% |  | 0.45 | 0.26 | 0.78 | 0.0049 | 16.8% |
| PC ae C32:1 | 0.80 | 0.65 | 0.98 | 0.1037 |  | 0.79 | 0.62 | 1.00 | 0.0483 | 74.9% |  | 0.83 | 0.56 | 1.25 | 0.3827 | 25.1% |
| PC ae C32:2 | 0.73 | 0.59 | 0.90 | 0.0307 |  | 0.72 | 0.57 | 0.92 | 0.0079 | 77.0% |  | 0.74 | 0.48 | 1.15 | 0.1857 | 23.0% |
| PC ae C34:0 | 0.72 | 0.58 | 0.90 | 0.0353 |  | 0.73 | 0.57 | 0.94 | 0.0150 | 75.9% |  | 0.70 | 0.45 | 1.10 | 0.1201 | 24.1% |
| PC ae C34:1 | 0.79 | 0.64 | 0.98 | 0.0996 |  | 0.83 | 0.65 | 1.05 | 0.1208 | 77.8% |  | 0.67 | 0.43 | 1.05 | 0.0797 | 22.2% |
| PC ae C34:2 | 0.92 | 0.75 | 1.13 | 0.5541 |  | 0.99 | 0.78 | 1.25 | 0.9054 | 77.8% |  | 0.72 | 0.46 | 1.11 | 0.1403 | 22.2% |
| PC ae C34:3 | 0.86 | 0.70 | 1.05 | 0.2965 |  | 0.85 | 0.67 | 1.08 | 0.1764 | 74.5% |  | 0.88 | 0.58 | 1.32 | 0.5259 | 25.5% |
| PC ae C36:0 | 0.72 | 0.58 | 0.91 | 0.0353 |  | 0.71 | 0.55 | 0.91 | 0.0081 | 78.7% |  | 0.79 | 0.48 | 1.29 | 0.3401 | 21.3% |
| PC ae C36:1 | 0.73 | 0.59 | 0.91 | 0.0353 |  | 0.80 | 0.63 | 1.03 | 0.0781 | 79.7% |  | 0.52 | 0.32 | 0.84 | 0.0082 | 20.3% |
| PC ae C36:2 | 0.71 | 0.56 | 0.88 | 0.0282 |  | 0.77 | 0.60 | 0.99 | 0.0452 | 79.9% |  | 0.49 | 0.30 | 0.82 | 0.0064 | 20.1% |
| PC ae C36:3 | 0.89 | 0.73 | 1.09 | 0.3993 |  | 0.95 | 0.75 | 1.19 | 0.6265 | 76.6% |  | 0.74 | 0.49 | 1.12 | 0.1526 | 23.4% |
| PC ae C36:4 | 1.04 | 0.86 | 1.27 | 0.7619 |  | 1.06 | 0.85 | 1.32 | 0.6264 | 77.9% |  | 1.00 | 0.66 | 1.52 | 0.9856 | 22.1% |
| PC ae C36:5 | 0.91 | 0.74 | 1.11 | 0.5029 |  | 0.90 | 0.72 | 1.13 | 0.3771 | 76.6% |  | 0.94 | 0.62 | 1.42 | 0.7609 | 23.4% |
| PC ae C38:0 | 0.68 | 0.54 | 0.85 | 0.0196 |  | 0.69 | 0.54 | 0.90 | 0.0053 | 76.5% |  | 0.64 | 0.40 | 1.02 | 0.0596 | 23.5% |
| PC ae C38:1 | 0.94 | 0.78 | 1.15 | 0.6657 |  | 0.95 | 0.76 | 1.19 | 0.6287 | 75.4% |  | 0.93 | 0.63 | 1.38 | 0.7321 | 24.6% |
| PC ae C38:2 | 0.75 | 0.60 | 0.93 | 0.0474 |  | 0.77 | 0.61 | 0.99 | 0.0407 | 76.6% |  | 0.67 | 0.43 | 1.05 | 0.0790 | 23.4% |
| PC ae C38:3 | 0.82 | 0.67 | 1.02 | 0.2013 |  | 0.94 | 0.74 | 1.19 | 0.6083 | 80.7% |  | 0.48 | 0.29 | 0.77 | 0.0025 | 19.3% |
| PC ae C38:4 | 0.90 | 0.73 | 1.10 | 0.4563 |  | 0.95 | 0.76 | 1.20 | 0.6725 | 80.7% |  | 0.71 | 0.44 | 1.13 | 0.1451 | 19.3% |
| PC ae C38:5 | 0.92 | 0.75 | 1.12 | 0.5248 |  | 0.89 | 0.71 | 1.12 | 0.3295 | 77.8% |  | 1.00 | 0.65 | 1.53 | 0.9971 | 22.2% |
| PC ae C38:6 | 0.78 | 0.63 | 0.96 | 0.0798 |  | 0.78 | 0.61 | 0.99 | 0.0419 | 76.8% |  | 0.77 | 0.49 | 1.20 | 0.2541 | 23.2% |
| PC ae C40:1 | 0.70 | 0.56 | 0.87 | 0.0196 |  | 0.71 | 0.55 | 0.90 | 0.0058 | 77.2% |  | 0.67 | 0.42 | 1.05 | 0.0807 | 22.8% |
| PC ae C40:2 | 0.73 | 0.58 | 0.91 | 0.0353 |  | 0.78 | 0.60 | 1.00 | 0.0526 | 76.6% |  | 0.59 | 0.37 | 0.93 | 0.0243 | 23.4% |
| PC ae C40:3 | 0.78 | 0.63 | 0.96 | 0.0815 |  | 0.83 | 0.65 | 1.05 | 0.1184 | 80.4% |  | 0.61 | 0.38 | 0.98 | 0.0428 | 19.6% |
| PC ae C40:4 | 0.77 | 0.61 | 0.96 | 0.0792 |  | 0.78 | 0.61 | 1.00 | 0.0536 | 77.7% |  | 0.72 | 0.45 | 1.14 | 0.1632 | 22.3% |
| PC ae C40:5 | 0.73 | 0.58 | 0.91 | 0.0353 |  | 0.71 | 0.55 | 0.91 | 0.0079 | 76.8% |  | 0.81 | 0.51 | 1.28 | 0.3605 | 23.2% |
| PC ae C40:6 | 0.66 | 0.52 | 0.82 | 0.0181 |  | 0.65 | 0.50 | 0.84 | 0.0012 | 74.9% |  | 0.67 | 0.43 | 1.05 | 0.0807 | 25.1% |
| PC ae C42:1 | 0.84 | 0.68 | 1.03 | 0.2510 |  | 0.84 | 0.67 | 1.07 | 0.1521 | 78.6% |  | 0.83 | 0.53 | 1.30 | 0.4050 | 21.4% |
| PC ae C42:2 | 0.68 | 0.54 | 0.84 | 0.0181 |  | 0.62 | 0.48 | 0.81 | 0.0004 | 70.2% |  | 0.82 | 0.55 | 1.22 | 0.3276 | 29.8% |
| PC ae C42:3 | 0.67 | 0.53 | 0.83 | 0.0181 |  | 0.67 | 0.52 | 0.86 | 0.0015 | 78.3% |  | 0.67 | 0.41 | 1.07 | 0.0917 | 21.7% |
| PC ae C42:4 | 0.88 | 0.71 | 1.09 | 0.3898 |  | 0.89 | 0.69 | 1.14 | 0.3474 | 76.6% |  | 0.86 | 0.55 | 1.34 | 0.5005 | 23.4% |
| PC ae C42:5 | 0.84 | 0.68 | 1.05 | 0.2926 |  | 0.81 | 0.63 | 1.04 | 0.0990 | 75.1% |  | 0.96 | 0.62 | 1.48 | 0.8421 | 24.9% |
| PC ae C44:3 | 0.88 | 0.72 | 1.08 | 0.3669 |  | 0.90 | 0.72 | 1.13 | 0.3726 | 78.9% |  | 0.82 | 0.53 | 1.27 | 0.3756 | 21.1% |
| PC ae C44:4 | 0.87 | 0.71 | 1.07 | 0.3315 |  | 0.85 | 0.67 | 1.07 | 0.1651 | 75.5% |  | 0.94 | 0.62 | 1.43 | 0.7862 | 24.5% |
| PC ae C44:5 | 0.92 | 0.76 | 1.13 | 0.5726 |  | 0.90 | 0.72 | 1.14 | 0.3854 | 77.4% |  | 1.00 | 0.65 | 1.53 | 0.9957 | 22.6% |
| PC ae C44:6 | 0.91 | 0.74 | 1.12 | 0.5248 |  | 0.85 | 0.67 | 1.07 | 0.1674 | 77.0% |  | 1.18 | 0.76 | 1.81 | 0.4623 | 23.0% |
| **lyso-Phosphatidylcholines** | | |  |  |  |  |  |  |  |  |  |  |  |  |  |  |
| lysoPC a C14:0 | 0.91 | 0.74 | 1.12 | 0.5248 |  | 0.94 | 0.74 | 1.19 | 0.6169 | 76.8% |  | 0.82 | 0.53 | 1.26 | 0.3658 | 23.2% |
| lysoPC a C16:0 | 0.82 | 0.65 | 1.02 | 0.1931 |  | 0.77 | 0.60 | 0.99 | 0.0421 | 75.8% |  | 0.98 | 0.62 | 1.52 | 0.9133 | 24.2% |
| lysoPC a C16:1 | 0.82 | 0.64 | 1.05 | 0.2729 |  | 0.77 | 0.58 | 1.02 | 0.0641 | 74.0% |  | 1.00 | 0.62 | 1.60 | 0.9927 | 26.0% |
| lysoPC a C17:0 | 0.73 | 0.58 | 0.91 | 0.0353 |  | 0.70 | 0.54 | 0.90 | 0.0062 | 74.3% |  | 0.82 | 0.53 | 1.27 | 0.3767 | 25.7% |
| lysoPC a C18:0 | 0.81 | 0.65 | 0.99 | 0.1188 |  | 0.76 | 0.60 | 0.98 | 0.0302 | 72.1% |  | 0.92 | 0.62 | 1.36 | 0.6848 | 27.9% |
| lysoPC a C18:1 | 0.79 | 0.63 | 0.99 | 0.1188 |  | 0.68 | 0.52 | 0.88 | 0.0037 | 75.9% |  | 1.26 | 0.79 | 2.00 | 0.3334 | 24.1% |
| lysoPC a C18:2 | 0.87 | 0.70 | 1.08 | 0.3409 |  | 0.78 | 0.61 | 1.00 | 0.0476 | 74.8% |  | 1.20 | 0.78 | 1.84 | 0.4040 | 25.2% |
| lysoPC a C20:3 | 0.87 | 0.70 | 1.08 | 0.3488 |  | 0.88 | 0.69 | 1.13 | 0.3071 | 79.5% |  | 0.83 | 0.51 | 1.35 | 0.4462 | 20.5% |
| lysoPC a C20:4 | 0.92 | 0.74 | 1.14 | 0.5851 |  | 0.84 | 0.65 | 1.07 | 0.1506 | 77.5% |  | 1.29 | 0.82 | 2.05 | 0.2681 | 22.5% |
| lysoPC a C28:1 | 0.87 | 0.71 | 1.08 | 0.3447 |  | 0.92 | 0.73 | 1.16 | 0.4767 | 80.9% |  | 0.70 | 0.43 | 1.13 | 0.1405 | 19.1% |
| **Sphingomyelins** |  |  |  |  |  |  |  |  |  |  |  |  |  |  |  |  |
| SM C16:0 | 1.00 | 0.82 | 1.22 | 0.9983 |  | 1.00 | 0.80 | 1.25 | 0.9942 | 80.0% |  | 1.02 | 0.66 | 1.59 | 0.9267 | 20.0% |
| SM C16:1 | 0.98 | 0.80 | 1.21 | 0.9230 |  | 1.00 | 0.80 | 1.26 | 0.9806 | 81.1% |  | 0.91 | 0.57 | 1.45 | 0.6823 | 18.9% |
| SM C18:0 | 1.03 | 0.84 | 1.27 | 0.8578 |  | 1.15 | 0.91 | 1.45 | 0.2386 | 81.7% |  | 0.63 | 0.39 | 1.03 | 0.0643 | 18.3% |
| SM C18:1 | 0.95 | 0.77 | 1.17 | 0.7224 |  | 1.01 | 0.80 | 1.27 | 0.9677 | 80.6% |  | 0.74 | 0.46 | 1.20 | 0.2249 | 19.4% |
| SM C20:2 | 0.85 | 0.69 | 1.06 | 0.2995 |  | 0.89 | 0.70 | 1.14 | 0.3654 | 77.6% |  | 0.73 | 0.46 | 1.14 | 0.1647 | 22.4% |
| SM C24:0 | 1.03 | 0.84 | 1.25 | 0.8721 |  | 1.09 | 0.87 | 1.37 | 0.4428 | 76.8% |  | 0.83 | 0.55 | 1.26 | 0.3853 | 23.2% |
| SM C24:1 | 1.01 | 0.83 | 1.23 | 0.9756 |  | 1.03 | 0.83 | 1.28 | 0.7874 | 79.9% |  | 0.93 | 0.60 | 1.43 | 0.7278 | 20.1% |
| SM C26:1 | 1.04 | 0.85 | 1.28 | 0.7788 |  | 1.02 | 0.81 | 1.29 | 0.8413 | 77.2% |  | 1.10 | 0.72 | 1.69 | 0.6515 | 22.8% |
| SM (OH) C14:1 | 0.85 | 0.68 | 1.05 | 0.2965 |  | 0.95 | 0.75 | 1.20 | 0.6590 | 84.3% |  | 0.46 | 0.27 | 0.79 | 0.0051 | 15.7% |
| SM (OH) C16:1 | 0.87 | 0.70 | 1.07 | 0.3409 |  | 0.95 | 0.75 | 1.20 | 0.6363 | 84.1% |  | 0.55 | 0.32 | 0.94 | 0.0285 | 15.9% |
| SM (OH) C22:1 | 0.91 | 0.74 | 1.11 | 0.4898 |  | 0.99 | 0.79 | 1.25 | 0.9303 | 80.8% |  | 0.62 | 0.39 | 0.99 | 0.0474 | 19.2% |
| SM (OH) C22:2 | 0.87 | 0.71 | 1.08 | 0.3488 |  | 0.96 | 0.77 | 1.20 | 0.7201 | 85.1% |  | 0.52 | 0.30 | 0.89 | 0.0171 | 14.9% |
| SM (OH) C24:1 | 0.99 | 0.81 | 1.21 | 0.9855 |  | 1.03 | 0.82 | 1.29 | 0.7889 | 78.2% |  | 0.87 | 0.57 | 1.34 | 0.5286 | 21.8% |

a, acyl; AC, acylcarnitines; e, alkyl; LCL, lower 95% confidence limit; OR, odds ratio; PC, phosphatidylcholines; SM, sphingomyelin; UCL, upper 95% confidence limit
