# Supplementary material for: Specific Metabolic Markers Are Associated with Future Waist-Gaining Phenotype in Women
Source: PLoS One. 2016 Jun 20;11(6):e0157733. doi: 10.1371/journal.pone.0157733 (PMC4920591; doi:10.1371/journal.pone.0157733)
Supplement: S4 Table — (DOCX) [file pone.0157733.s004.docx]

Table S4 Association of metabolites with hip-gaining phenotype in women in the combined fixed-effect meta-analysis and by specific study

|  | **overall** | | | |  | **EPIC-Potsdam** | | | | |  | **KORA** | | | | |
| --- | --- | --- | --- | --- | --- | --- | --- | --- | --- | --- | --- | --- | --- | --- | --- | --- |
| **Amino Acids** | **OR** | **LCL** | **UCL** | **FDR p** |  | **OR** | **LCL** | **UCL** | **uncorrected  p value** | **meta weight** |  | **OR** | **LCL** | **UCL** | **uncorrected  p value** | **meta weight** |
| Arg | 0.85 | 0.68 | 1.06 | 0.8165 |  | 0.91 | 0.71 | 1.16 | 0.4404 | 78.3% |  | 0.66 | 0.41 | 1.06 | 0.0856 | 21.7% |
| Gln | 1.07 | 0.87 | 1.32 | 0.8277 |  | 1.03 | 0.80 | 1.31 | 0.8368 | 73.9% |  | 1.20 | 0.79 | 1.82 | 0.3862 | 26.1% |
| Gly | 1.06 | 0.86 | 1.31 | 0.8277 |  | 1.08 | 0.84 | 1.38 | 0.5589 | 72.4% |  | 1.02 | 0.68 | 1.53 | 0.9257 | 27.6% |
| His | 1.01 | 0.82 | 1.25 | 0.9697 |  | 0.99 | 0.77 | 1.27 | 0.9132 | 71.6% |  | 1.07 | 0.72 | 1.59 | 0.7405 | 28.4% |
| Met | 1.00 | 0.81 | 1.23 | 0.9939 |  | 1.00 | 0.79 | 1.26 | 0.9870 | 76.0% |  | 1.00 | 0.66 | 1.52 | 0.9885 | 24.0% |
| Orn | 0.90 | 0.73 | 1.12 | 0.8165 |  | 0.93 | 0.72 | 1.19 | 0.5645 | 72.5% |  | 0.84 | 0.56 | 1.26 | 0.3941 | 27.5% |
| Phe | 0.92 | 0.75 | 1.14 | 0.8165 |  | 0.99 | 0.78 | 1.26 | 0.9507 | 76.2% |  | 0.74 | 0.48 | 1.14 | 0.1679 | 23.8% |
| Pro | 1.15 | 0.92 | 1.43 | 0.8165 |  | 1.20 | 0.93 | 1.53 | 0.1565 | 79.1% |  | 0.99 | 0.61 | 1.60 | 0.9671 | 20.9% |
| Ser | 1.14 | 0.92 | 1.41 | 0.8165 |  | 1.03 | 0.79 | 1.34 | 0.8318 | 67.0% |  | 1.39 | 0.96 | 2.01 | 0.0852 | 33.0% |
| Thr | 1.03 | 0.82 | 1.29 | 0.9044 |  | 0.91 | 0.69 | 1.19 | 0.4843 | 69.7% |  | 1.36 | 0.90 | 2.05 | 0.1430 | 30.3% |
| Trp | 0.97 | 0.79 | 1.20 | 0.8813 |  | 1.00 | 0.79 | 1.28 | 0.9831 | 76.4% |  | 0.87 | 0.57 | 1.35 | 0.5415 | 23.6% |
| Tyr | 1.05 | 0.86 | 1.29 | 0.8277 |  | 1.08 | 0.86 | 1.36 | 0.5094 | 76.1% |  | 0.97 | 0.65 | 1.46 | 0.8979 | 23.9% |
| Val | 1.10 | 0.89 | 1.36 | 0.8165 |  | 1.18 | 0.93 | 1.50 | 0.1841 | 76.1% |  | 0.89 | 0.58 | 1.37 | 0.6074 | 23.9% |
| **Hexose** |  |  |  |  |  |  |  |  |  |  |  |  |  |  |  |  |
| H1 | 1.04 | 0.86 | 1.27 | 0.8277 |  | 1.06 | 0.85 | 1.31 | 0.6091 | 81.2% |  | 0.99 | 0.64 | 1.55 | 0.9748 | 18.8% |
| **Acylcarnitines** |  |  |  |  |  |  |  |  |  |  |  |  |  |  |  |  |
| C0 | 1.22 | 0.98 | 1.53 | 0.8165 |  | 1.18 | 0.91 | 1.53 | 0.2160 | 74.0% |  | 1.35 | 0.87 | 2.09 | 0.1846 | 26.0% |
| C2 | 1.09 | 0.87 | 1.35 | 0.8165 |  | 1.08 | 0.83 | 1.39 | 0.5742 | 72.8% |  | 1.11 | 0.73 | 1.69 | 0.6298 | 27.2% |
| C3 | 1.15 | 0.93 | 1.42 | 0.8165 |  | 1.12 | 0.88 | 1.44 | 0.3516 | 73.6% |  | 1.21 | 0.80 | 1.83 | 0.3587 | 26.4% |
| C5-OH (C3-DC-M) | 1.07 | 0.88 | 1.31 | 0.8277 |  | 1.12 | 0.89 | 1.41 | 0.3212 | 75.0% |  | 0.93 | 0.62 | 1.38 | 0.7133 | 25.0% |
| C7-DC | 1.16 | 0.93 | 1.44 | 0.8165 |  | 1.27 | 1.00 | 1.61 | 0.0491 | 83.3% |  | 0.73 | 0.43 | 1.25 | 0.2557 | 16.7% |
| C9 | 1.24 | 1.00 | 1.54 | 0.8165 |  | 1.31 | 1.03 | 1.66 | 0.0304 | 79.9% |  | 1.01 | 0.62 | 1.63 | 0.9717 | 20.1% |
| C10 | 1.18 | 0.99 | 1.40 | 0.8165 |  | 1.15 | 0.93 | 1.42 | 0.1868 | 69.9% |  | 1.23 | 0.90 | 1.70 | 0.1941 | 30.1% |
| C10:2 | 1.01 | 0.83 | 1.24 | 0.9697 |  | 1.08 | 0.86 | 1.36 | 0.4967 | 80.5% |  | 0.77 | 0.48 | 1.21 | 0.2572 | 19.5% |
| C14:1 | 1.10 | 0.89 | 1.35 | 0.8165 |  | 1.09 | 0.85 | 1.40 | 0.5168 | 71.4% |  | 1.12 | 0.75 | 1.66 | 0.5808 | 28.6% |
| C14:2 | 1.22 | 0.99 | 1.51 | 0.8165 |  | 1.26 | 0.99 | 1.61 | 0.0656 | 74.2% |  | 1.12 | 0.74 | 1.70 | 0.5892 | 25.8% |
| C16 | 1.06 | 0.85 | 1.33 | 0.8277 |  | 1.08 | 0.84 | 1.41 | 0.5463 | 74.0% |  | 1.00 | 0.65 | 1.55 | 0.9945 | 26.0% |
| C18 | 0.98 | 0.78 | 1.21 | 0.9044 |  | 0.92 | 0.71 | 1.19 | 0.5163 | 73.8% |  | 1.16 | 0.76 | 1.77 | 0.5015 | 26.2% |
| C18:1 | 1.05 | 0.84 | 1.31 | 0.8277 |  | 1.00 | 0.77 | 1.30 | 0.9929 | 70.6% |  | 1.19 | 0.79 | 1.79 | 0.4057 | 29.4% |
| C18:2 | 1.05 | 0.84 | 1.31 | 0.8277 |  | 0.98 | 0.76 | 1.26 | 0.8555 | 76.4% |  | 1.33 | 0.84 | 2.11 | 0.2251 | 23.6% |
| **diacyl-Phosphatidylcholines** | |  |  |  |  |  |  |  |  |  |  |  |  |  |  |  |
| PC aa C28:1 | 1.01 | 0.81 | 1.25 | 0.9832 |  | 0.97 | 0.75 | 1.26 | 0.8299 | 71.8% |  | 1.10 | 0.73 | 1.66 | 0.6588 | 28.2% |
| PC aa C30:0 | 0.93 | 0.75 | 1.16 | 0.8277 |  | 0.94 | 0.73 | 1.21 | 0.6271 | 76.3% |  | 0.91 | 0.58 | 1.43 | 0.6824 | 23.7% |
| PC aa C32:0 | 0.99 | 0.80 | 1.23 | 0.9697 |  | 0.91 | 0.71 | 1.17 | 0.4633 | 76.6% |  | 1.30 | 0.83 | 2.03 | 0.2542 | 23.4% |
| PC aa C32:1 | 0.92 | 0.74 | 1.14 | 0.8165 |  | 0.93 | 0.73 | 1.19 | 0.5533 | 80.9% |  | 0.88 | 0.53 | 1.45 | 0.6065 | 19.1% |
| PC aa C32:2 | 0.86 | 0.69 | 1.06 | 0.8165 |  | 0.90 | 0.70 | 1.16 | 0.4306 | 75.1% |  | 0.73 | 0.47 | 1.12 | 0.1516 | 24.9% |
| PC aa C32:3 | 1.04 | 0.84 | 1.29 | 0.8344 |  | 0.95 | 0.74 | 1.22 | 0.7075 | 74.4% |  | 1.33 | 0.87 | 2.03 | 0.1848 | 25.6% |
| PC aa C34:1 | 0.89 | 0.72 | 1.11 | 0.8165 |  | 0.86 | 0.67 | 1.10 | 0.2333 | 74.5% |  | 1.01 | 0.66 | 1.55 | 0.9702 | 25.5% |
| PC aa C34:2 | 0.91 | 0.74 | 1.12 | 0.8165 |  | 0.85 | 0.67 | 1.09 | 0.1984 | 71.9% |  | 1.07 | 0.72 | 1.58 | 0.7459 | 28.1% |
| PC aa C34:3 | 0.90 | 0.73 | 1.11 | 0.8165 |  | 0.89 | 0.69 | 1.13 | 0.3278 | 74.4% |  | 0.95 | 0.63 | 1.44 | 0.8138 | 25.6% |
| PC aa C34:4 | 0.89 | 0.72 | 1.10 | 0.8165 |  | 0.95 | 0.74 | 1.22 | 0.6746 | 75.4% |  | 0.73 | 0.47 | 1.12 | 0.1483 | 24.6% |
| PC aa C36:0 | 1.08 | 0.86 | 1.34 | 0.8277 |  | 0.93 | 0.72 | 1.19 | 0.5645 | 76.0% |  | 1.71 | 1.10 | 2.67 | 0.0179 | 24.0% |
| PC aa C36:1 | 0.83 | 0.67 | 1.03 | 0.8165 |  | 0.82 | 0.64 | 1.05 | 0.1076 | 73.9% |  | 0.88 | 0.58 | 1.33 | 0.5397 | 26.1% |
| PC aa C36:2 | 0.85 | 0.69 | 1.05 | 0.8165 |  | 0.80 | 0.62 | 1.03 | 0.0797 | 69.0% |  | 0.97 | 0.67 | 1.41 | 0.8830 | 31.0% |
| PC aa C36:3 | 0.92 | 0.75 | 1.14 | 0.8165 |  | 0.92 | 0.72 | 1.17 | 0.4786 | 72.8% |  | 0.95 | 0.64 | 1.41 | 0.7853 | 27.2% |
| PC aa C36:4 | 0.91 | 0.73 | 1.13 | 0.8165 |  | 0.90 | 0.70 | 1.15 | 0.3891 | 74.6% |  | 0.95 | 0.62 | 1.46 | 0.8326 | 25.4% |
| PC aa C36:5 | 0.97 | 0.79 | 1.19 | 0.8813 |  | 0.90 | 0.71 | 1.14 | 0.3749 | 73.2% |  | 1.21 | 0.82 | 1.79 | 0.3454 | 26.8% |
| PC aa C36:6 | 0.92 | 0.75 | 1.14 | 0.8165 |  | 0.88 | 0.69 | 1.12 | 0.3092 | 75.9% |  | 1.07 | 0.70 | 1.63 | 0.7638 | 24.1% |
| PC aa C38:0 | 1.04 | 0.83 | 1.30 | 0.8344 |  | 0.86 | 0.66 | 1.12 | 0.2669 | 69.8% |  | 1.62 | 1.08 | 2.44 | 0.0208 | 30.2% |
| PC aa C38:1 | 1.12 | 0.90 | 1.39 | 0.8165 |  | 1.06 | 0.83 | 1.36 | 0.6253 | 76.5% |  | 1.33 | 0.85 | 2.07 | 0.2174 | 23.5% |
| PC aa C38:3 | 0.84 | 0.68 | 1.05 | 0.8165 |  | 0.86 | 0.67 | 1.11 | 0.2569 | 73.5% |  | 0.78 | 0.51 | 1.20 | 0.2569 | 26.5% |
| PC aa C38:4 | 0.86 | 0.70 | 1.07 | 0.8165 |  | 0.87 | 0.68 | 1.11 | 0.2665 | 75.0% |  | 0.84 | 0.55 | 1.29 | 0.4281 | 25.0% |
| PC aa C38:5 | 0.94 | 0.76 | 1.16 | 0.8277 |  | 0.89 | 0.70 | 1.14 | 0.3535 | 75.4% |  | 1.10 | 0.72 | 1.67 | 0.6713 | 24.6% |
| PC aa C38:6 | 0.88 | 0.71 | 1.10 | 0.8165 |  | 0.79 | 0.61 | 1.02 | 0.0678 | 73.9% |  | 1.21 | 0.79 | 1.87 | 0.3740 | 26.1% |
| PC aa C40:2 | 1.04 | 0.84 | 1.28 | 0.8344 |  | 0.99 | 0.77 | 1.27 | 0.9340 | 74.1% |  | 1.19 | 0.78 | 1.80 | 0.4165 | 25.9% |
| PC aa C40:3 | 1.06 | 0.84 | 1.33 | 0.8277 |  | 0.98 | 0.75 | 1.28 | 0.8874 | 72.0% |  | 1.28 | 0.83 | 1.96 | 0.2661 | 28.0% |
| PC aa C40:4 | 0.86 | 0.70 | 1.06 | 0.8165 |  | 0.91 | 0.72 | 1.15 | 0.4097 | 80.5% |  | 0.70 | 0.44 | 1.13 | 0.1480 | 19.5% |
| PC aa C40:5 | 0.79 | 0.64 | 0.98 | 0.8165 |  | 0.83 | 0.65 | 1.06 | 0.1326 | 78.0% |  | 0.67 | 0.42 | 1.07 | 0.0962 | 22.0% |
| PC aa C40:6 | 0.79 | 0.63 | 0.99 | 0.8165 |  | 0.71 | 0.55 | 0.93 | 0.0125 | 70.8% |  | 1.01 | 0.67 | 1.52 | 0.9767 | 29.2% |
| PC aa C42:0 | 1.11 | 0.88 | 1.39 | 0.8165 |  | 1.00 | 0.77 | 1.31 | 0.9773 | 71.9% |  | 1.42 | 0.93 | 2.17 | 0.1043 | 28.1% |
| PC aa C42:1 | 1.05 | 0.84 | 1.32 | 0.8277 |  | 0.97 | 0.74 | 1.27 | 0.8170 | 72.8% |  | 1.30 | 0.84 | 2.02 | 0.2336 | 27.2% |
| PC aa C42:2 | 1.09 | 0.89 | 1.34 | 0.8165 |  | 0.96 | 0.76 | 1.21 | 0.7429 | 79.8% |  | 1.79 | 1.13 | 2.84 | 0.0136 | 20.2% |
| PC aa C42:4 | 0.91 | 0.73 | 1.14 | 0.8165 |  | 0.81 | 0.62 | 1.06 | 0.1173 | 68.6% |  | 1.18 | 0.80 | 1.76 | 0.4013 | 31.4% |
| PC aa C42:5 | 0.92 | 0.74 | 1.15 | 0.8277 |  | 0.92 | 0.71 | 1.19 | 0.5068 | 72.8% |  | 0.95 | 0.62 | 1.44 | 0.7946 | 27.2% |
| PC aa C42:6 | 0.95 | 0.77 | 1.17 | 0.8277 |  | 0.93 | 0.73 | 1.18 | 0.5522 | 75.0% |  | 1.03 | 0.68 | 1.56 | 0.8945 | 25.0% |
| **acyl-alkyl-Phosphatidylcholines** | |  |  |  |  |  |  |  |  |  |  |  |  |  |  |  |
| PC ae C30:0 | 1.16 | 0.93 | 1.46 | 0.8165 |  | 1.11 | 0.85 | 1.45 | 0.4527 | 73.5% |  | 1.34 | 0.86 | 2.09 | 0.1983 | 26.5% |
| PC ae C30:2 | 1.06 | 0.84 | 1.33 | 0.8277 |  | 1.01 | 0.77 | 1.33 | 0.9441 | 72.3% |  | 1.19 | 0.76 | 1.85 | 0.4423 | 27.7% |
| PC ae C32:1 | 1.14 | 0.91 | 1.42 | 0.8165 |  | 0.98 | 0.75 | 1.28 | 0.8736 | 70.1% |  | 1.61 | 1.07 | 2.41 | 0.0211 | 29.9% |
| PC ae C32:2 | 1.25 | 1.00 | 1.56 | 0.8165 |  | 1.04 | 0.80 | 1.35 | 0.7749 | 71.5% |  | 2.00 | 1.32 | 3.03 | 0.0012 | 28.5% |
| PC ae C34:0 | 1.11 | 0.89 | 1.39 | 0.8165 |  | 1.08 | 0.83 | 1.39 | 0.5778 | 74.6% |  | 1.23 | 0.79 | 1.91 | 0.3521 | 25.4% |
| PC ae C34:1 | 1.19 | 0.95 | 1.49 | 0.8165 |  | 1.09 | 0.84 | 1.41 | 0.5432 | 72.9% |  | 1.52 | 0.99 | 2.34 | 0.0539 | 27.1% |
| PC ae C34:2 | 1.13 | 0.91 | 1.42 | 0.8165 |  | 0.93 | 0.72 | 1.20 | 0.5708 | 75.6% |  | 2.10 | 1.33 | 3.30 | 0.0014 | 24.4% |
| PC ae C34:3 | 1.14 | 0.91 | 1.44 | 0.8165 |  | 0.90 | 0.69 | 1.18 | 0.4482 | 71.6% |  | 2.09 | 1.36 | 3.22 | 0.0008 | 28.4% |
| PC ae C36:0 | 1.03 | 0.83 | 1.27 | 0.8991 |  | 0.97 | 0.76 | 1.25 | 0.8177 | 75.3% |  | 1.22 | 0.79 | 1.88 | 0.3751 | 24.7% |
| PC ae C36:1 | 1.05 | 0.84 | 1.31 | 0.8277 |  | 1.02 | 0.79 | 1.32 | 0.8952 | 74.2% |  | 1.15 | 0.74 | 1.78 | 0.5282 | 25.8% |
| PC ae C36:2 | 1.16 | 0.93 | 1.45 | 0.8165 |  | 1.07 | 0.83 | 1.39 | 0.5901 | 74.3% |  | 1.46 | 0.94 | 2.26 | 0.0940 | 25.7% |
| PC ae C36:3 | 1.09 | 0.88 | 1.36 | 0.8165 |  | 0.90 | 0.70 | 1.17 | 0.4419 | 71.5% |  | 1.75 | 1.16 | 2.64 | 0.0078 | 28.5% |
| PC ae C36:4 | 1.07 | 0.87 | 1.32 | 0.8277 |  | 1.00 | 0.78 | 1.28 | 0.9965 | 71.7% |  | 1.27 | 0.86 | 1.89 | 0.2314 | 28.3% |
| PC ae C36:5 | 1.06 | 0.86 | 1.31 | 0.8277 |  | 0.95 | 0.74 | 1.21 | 0.6673 | 71.6% |  | 1.41 | 0.95 | 2.09 | 0.0848 | 28.4% |
| PC ae C38:0 | 1.00 | 0.80 | 1.24 | 0.9923 |  | 0.89 | 0.69 | 1.14 | 0.3433 | 75.2% |  | 1.43 | 0.92 | 2.21 | 0.1119 | 24.8% |
| PC ae C38:1 | 0.91 | 0.73 | 1.14 | 0.8165 |  | 0.90 | 0.69 | 1.16 | 0.4033 | 73.5% |  | 0.96 | 0.62 | 1.47 | 0.8355 | 26.5% |
| PC ae C38:2 | 1.09 | 0.87 | 1.36 | 0.8165 |  | 1.10 | 0.85 | 1.42 | 0.4886 | 73.0% |  | 1.07 | 0.70 | 1.64 | 0.7455 | 27.0% |
| PC ae C38:3 | 1.11 | 0.90 | 1.37 | 0.8165 |  | 1.11 | 0.88 | 1.41 | 0.3853 | 75.7% |  | 1.10 | 0.72 | 1.68 | 0.6442 | 24.3% |
| PC ae C38:4 | 1.08 | 0.86 | 1.34 | 0.8277 |  | 0.99 | 0.77 | 1.28 | 0.9409 | 73.2% |  | 1.35 | 0.88 | 2.06 | 0.1650 | 26.8% |
| PC ae C38:5 | 1.15 | 0.93 | 1.41 | 0.8165 |  | 1.01 | 0.79 | 1.29 | 0.9353 | 70.6% |  | 1.56 | 1.06 | 2.29 | 0.0226 | 29.4% |
| PC ae C38:6 | 1.07 | 0.86 | 1.33 | 0.8277 |  | 0.93 | 0.72 | 1.19 | 0.5461 | 74.3% |  | 1.62 | 1.06 | 2.47 | 0.0270 | 25.7% |
| PC ae C40:1 | 0.95 | 0.76 | 1.19 | 0.8277 |  | 0.87 | 0.67 | 1.12 | 0.2730 | 75.1% |  | 1.26 | 0.81 | 1.97 | 0.3020 | 24.9% |
| PC ae C40:2 | 1.10 | 0.89 | 1.36 | 0.8165 |  | 1.00 | 0.78 | 1.28 | 0.9955 | 73.7% |  | 1.43 | 0.94 | 2.15 | 0.0917 | 26.3% |
| PC ae C40:3 | 1.04 | 0.84 | 1.30 | 0.8344 |  | 0.93 | 0.72 | 1.21 | 0.5957 | 72.2% |  | 1.40 | 0.92 | 2.13 | 0.1118 | 27.8% |
| PC ae C40:4 | 1.09 | 0.87 | 1.37 | 0.8165 |  | 1.01 | 0.77 | 1.32 | 0.9541 | 71.5% |  | 1.32 | 0.87 | 2.02 | 0.1932 | 28.5% |
| PC ae C40:5 | 1.10 | 0.89 | 1.37 | 0.8165 |  | 0.99 | 0.77 | 1.27 | 0.9304 | 73.9% |  | 1.50 | 0.99 | 2.29 | 0.0573 | 26.1% |
| PC ae C40:6 | 1.10 | 0.88 | 1.38 | 0.8165 |  | 0.92 | 0.71 | 1.20 | 0.5269 | 72.2% |  | 1.75 | 1.14 | 2.68 | 0.0099 | 27.8% |
| PC ae C42:1 | 0.95 | 0.77 | 1.19 | 0.8277 |  | 0.94 | 0.73 | 1.21 | 0.6037 | 73.3% |  | 1.01 | 0.66 | 1.54 | 0.9560 | 26.7% |
| PC ae C42:2 | 0.87 | 0.70 | 1.08 | 0.8165 |  | 0.80 | 0.62 | 1.03 | 0.0831 | 74.2% |  | 1.09 | 0.72 | 1.67 | 0.6795 | 25.8% |
| PC ae C42:3 | 1.00 | 0.79 | 1.26 | 0.9939 |  | 0.85 | 0.65 | 1.11 | 0.2269 | 73.4% |  | 1.57 | 1.00 | 2.47 | 0.0479 | 26.6% |
| PC ae C42:4 | 1.12 | 0.89 | 1.42 | 0.8165 |  | 0.99 | 0.76 | 1.31 | 0.9565 | 72.2% |  | 1.55 | 1.00 | 2.42 | 0.0508 | 27.8% |
| PC ae C42:5 | 1.14 | 0.91 | 1.43 | 0.8165 |  | 1.04 | 0.80 | 1.36 | 0.7701 | 69.4% |  | 1.41 | 0.94 | 2.10 | 0.0961 | 30.6% |
| PC ae C44:3 | 0.86 | 0.68 | 1.08 | 0.8165 |  | 0.84 | 0.64 | 1.10 | 0.2039 | 68.5% |  | 0.91 | 0.61 | 1.37 | 0.6541 | 31.5% |
| PC ae C44:4 | 1.10 | 0.87 | 1.37 | 0.8165 |  | 1.06 | 0.81 | 1.39 | 0.6698 | 71.0% |  | 1.19 | 0.78 | 1.81 | 0.4170 | 29.0% |
| PC ae C44:5 | 1.16 | 0.93 | 1.46 | 0.8165 |  | 1.07 | 0.82 | 1.41 | 0.6147 | 69.3% |  | 1.39 | 0.92 | 2.09 | 0.1148 | 30.7% |
| PC ae C44:6 | 1.14 | 0.90 | 1.44 | 0.8165 |  | 1.01 | 0.77 | 1.33 | 0.9264 | 72.3% |  | 1.56 | 1.00 | 2.42 | 0.0492 | 27.7% |
| **lyso-Phosphatidylcholines** | |  |  |  |  |  |  |  |  |  |  |  |  |  |  |  |
| lysoPC a C14:0 | 0.94 | 0.76 | 1.17 | 0.8277 |  | 1.04 | 0.81 | 1.33 | 0.7703 | 77.8% |  | 0.68 | 0.43 | 1.07 | 0.0969 | 22.2% |
| lysoPC a C16:0 | 0.89 | 0.71 | 1.11 | 0.8165 |  | 0.89 | 0.69 | 1.15 | 0.3756 | 75.1% |  | 0.88 | 0.56 | 1.38 | 0.5708 | 24.9% |
| lysoPC a C16:1 | 0.96 | 0.80 | 1.16 | 0.8344 |  | 0.96 | 0.78 | 1.18 | 0.7150 | 83.1% |  | 0.98 | 0.62 | 1.55 | 0.9280 | 16.9% |
| lysoPC a C17:0 | 1.11 | 0.89 | 1.39 | 0.8165 |  | 1.12 | 0.87 | 1.46 | 0.3801 | 74.4% |  | 1.07 | 0.69 | 1.67 | 0.7585 | 25.6% |
| lysoPC a C18:0 | 0.83 | 0.67 | 1.03 | 0.8165 |  | 0.84 | 0.65 | 1.08 | 0.1683 | 73.2% |  | 0.81 | 0.54 | 1.22 | 0.3131 | 26.8% |
| lysoPC a C18:1 | 0.95 | 0.78 | 1.17 | 0.8277 |  | 0.93 | 0.74 | 1.16 | 0.5046 | 82.5% |  | 1.10 | 0.67 | 1.79 | 0.7034 | 17.5% |
| lysoPC a C18:2 | 0.96 | 0.76 | 1.21 | 0.8344 |  | 0.93 | 0.71 | 1.22 | 0.5951 | 74.1% |  | 1.04 | 0.66 | 1.64 | 0.8589 | 25.9% |
| lysoPC a C20:3 | 0.90 | 0.73 | 1.11 | 0.8165 |  | 0.94 | 0.75 | 1.18 | 0.5991 | 82.7% |  | 0.73 | 0.44 | 1.20 | 0.2146 | 17.3% |
| lysoPC a C20:4 | 0.94 | 0.76 | 1.17 | 0.8277 |  | 0.95 | 0.76 | 1.20 | 0.6899 | 86.4% |  | 0.85 | 0.47 | 1.53 | 0.5856 | 13.6% |
| lysoPC a C28:1 | 0.99 | 0.78 | 1.25 | 0.9697 |  | 0.95 | 0.72 | 1.27 | 0.7421 | 67.7% |  | 1.06 | 0.71 | 1.60 | 0.7703 | 32.3% |
| **Sphingomyelins** |  |  |  |  |  |  |  |  |  |  |  |  |  |  |  |  |
| SM C16:0 | 1.05 | 0.84 | 1.32 | 0.8277 |  | 0.90 | 0.70 | 1.17 | 0.4400 | 73.8% |  | 1.62 | 1.05 | 2.50 | 0.0307 | 26.2% |
| SM C16:1 | 1.16 | 0.92 | 1.46 | 0.8165 |  | 0.98 | 0.75 | 1.28 | 0.8785 | 72.2% |  | 1.80 | 1.16 | 2.78 | 0.0084 | 27.8% |
| SM C18:0 | 0.94 | 0.76 | 1.17 | 0.8277 |  | 0.86 | 0.67 | 1.11 | 0.2436 | 73.2% |  | 1.21 | 0.80 | 1.82 | 0.3753 | 26.8% |
| SM C18:1 | 1.06 | 0.85 | 1.32 | 0.8277 |  | 0.94 | 0.73 | 1.22 | 0.6328 | 71.6% |  | 1.43 | 0.95 | 2.15 | 0.0901 | 28.4% |
| SM C20:2 | 1.22 | 0.98 | 1.51 | 0.8165 |  | 1.08 | 0.84 | 1.39 | 0.5493 | 72.7% |  | 1.68 | 1.11 | 2.53 | 0.0138 | 27.3% |
| SM C24:0 | 0.79 | 0.64 | 0.98 | 0.8165 |  | 0.76 | 0.59 | 0.98 | 0.0338 | 72.1% |  | 0.88 | 0.59 | 1.32 | 0.5388 | 27.9% |
| SM C24:1 | 1.01 | 0.81 | 1.25 | 0.9697 |  | 0.91 | 0.71 | 1.17 | 0.4667 | 71.1% |  | 1.30 | 0.87 | 1.93 | 0.1980 | 28.9% |
| SM C26:1 | 0.96 | 0.77 | 1.19 | 0.8344 |  | 0.84 | 0.65 | 1.09 | 0.1832 | 72.0% |  | 1.36 | 0.90 | 2.05 | 0.1468 | 28.0% |
| SM (OH) C14:1 | 1.18 | 0.94 | 1.47 | 0.8165 |  | 1.09 | 0.84 | 1.42 | 0.5300 | 71.5% |  | 1.44 | 0.95 | 2.19 | 0.0860 | 28.5% |
| SM (OH) C16:1 | 1.14 | 0.92 | 1.41 | 0.8165 |  | 1.05 | 0.81 | 1.35 | 0.7283 | 73.0% |  | 1.43 | 0.95 | 2.16 | 0.0893 | 27.0% |
| SM (OH) C22:1 | 0.89 | 0.72 | 1.11 | 0.8165 |  | 0.87 | 0.68 | 1.12 | 0.2901 | 74.1% |  | 0.95 | 0.62 | 1.45 | 0.7975 | 25.9% |
| SM (OH) C22:2 | 1.07 | 0.85 | 1.34 | 0.8277 |  | 0.97 | 0.75 | 1.26 | 0.8162 | 74.5% |  | 1.42 | 0.91 | 2.22 | 0.1222 | 25.5% |
| SM (OH) C24:1 | 1.01 | 0.82 | 1.25 | 0.9697 |  | 0.99 | 0.77 | 1.27 | 0.9260 | 72.9% |  | 1.08 | 0.72 | 1.62 | 0.7032 | 27.1% |

a, acyl; AC, acylcarnitines; e, alkyl; LCL, lower 95% confidence limit; OR, odds ratio; PC, phosphatidylcholines; SM, sphingomyelin; UCL, upper 95% confidence limit
