# Supplementary material for: Specific Metabolic Markers Are Associated with Future Waist-Gaining Phenotype in Women
Source: PLoS One. 2016 Jun 20;11(6):e0157733. doi: 10.1371/journal.pone.0157733 (PMC4920591; doi:10.1371/journal.pone.0157733)
Supplement: S5 Table — (DOCX) [file pone.0157733.s005.docx]

Table S5 Association of metabolites with waist-gaining phenotype in men in the combined fixed-effect meta-analysis and by specific study

|  | **overall** | | | |  | **EPIC-Potsdam** | | | | |  | **KORA** | | | | |
| --- | --- | --- | --- | --- | --- | --- | --- | --- | --- | --- | --- | --- | --- | --- | --- | --- |
| **Amino Acids** | **OR** | **LCL** | **UCL** | **FDR p** |  | **OR** | **LCL** | **UCL** | **uncorrected  p value** | **meta weight** |  | **OR** | **LCL** | **UCL** | **uncorrected  p value** | **meta weight** |
| Arg | 1.10 | 0.87 | 1.40 | 0.6891 |  | 0.94 | 0.69 | 1.29 | 0.7113 | 58.0% |  | 1.37 | 0.95 | 1.97 | 0.0929 | 42.0% |
| Gln | 1.10 | 0.87 | 1.39 | 0.6891 |  | 0.93 | 0.69 | 1.27 | 0.6662 | 58.8% |  | 1.39 | 0.96 | 2.01 | 0.0792 | 41.2% |
| Gly | 0.91 | 0.68 | 1.22 | 0.7474 |  | 0.93 | 0.66 | 1.31 | 0.6791 | 72.8% |  | 0.86 | 0.49 | 1.51 | 0.6030 | 27.2% |
| His | 1.10 | 0.86 | 1.41 | 0.7158 |  | 0.90 | 0.65 | 1.24 | 0.5127 | 58.8% |  | 1.47 | 1.00 | 2.16 | 0.0509 | 41.2% |
| Met | 1.03 | 0.80 | 1.31 | 0.8786 |  | 0.85 | 0.61 | 1.18 | 0.3274 | 55.1% |  | 1.30 | 0.90 | 1.87 | 0.1671 | 44.9% |
| Orn | 1.13 | 0.88 | 1.45 | 0.6891 |  | 0.85 | 0.61 | 1.19 | 0.3439 | 58.9% |  | 1.69 | 1.14 | 2.50 | 0.0092 | 41.1% |
| Phe | 1.12 | 0.88 | 1.43 | 0.6891 |  | 1.07 | 0.79 | 1.46 | 0.6627 | 60.8% |  | 1.20 | 0.81 | 1.76 | 0.3664 | 39.2% |
| Pro | 1.06 | 0.84 | 1.35 | 0.7849 |  | 1.01 | 0.75 | 1.38 | 0.9279 | 59.6% |  | 1.14 | 0.79 | 1.66 | 0.4777 | 40.4% |
| Ser | 0.91 | 0.70 | 1.19 | 0.7326 |  | 0.76 | 0.54 | 1.05 | 0.0986 | 63.1% |  | 1.25 | 0.81 | 1.94 | 0.3111 | 36.9% |
| Thr | 0.79 | 0.60 | 1.04 | 0.6891 |  | 0.71 | 0.49 | 1.02 | 0.0649 | 55.8% |  | 0.91 | 0.60 | 1.37 | 0.6538 | 44.2% |
| Trp | 1.06 | 0.83 | 1.36 | 0.7871 |  | 0.92 | 0.68 | 1.26 | 0.6143 | 64.1% |  | 1.37 | 0.90 | 2.08 | 0.1386 | 35.9% |
| Tyr | 1.07 | 0.84 | 1.36 | 0.7785 |  | 0.91 | 0.66 | 1.24 | 0.5444 | 57.7% |  | 1.33 | 0.92 | 1.93 | 0.1254 | 42.3% |
| Val | 1.17 | 0.92 | 1.49 | 0.6891 |  | 1.01 | 0.74 | 1.37 | 0.9595 | 64.0% |  | 1.52 | 1.02 | 2.28 | 0.0413 | 36.0% |
| **Hexose** |  |  |  |  |  |  |  |  |  |  |  |  |  |  |  |  |
| H1 | 1.29 | 0.98 | 1.68 | 0.5684 |  | 1.33 | 0.97 | 1.84 | 0.0810 | 69.2% |  | 1.19 | 0.73 | 1.92 | 0.4864 | 30.8% |
| **Acylcarnitines** |  |  |  |  |  |  |  |  |  |  |  |  |  |  |  |  |
| C0 | 1.15 | 0.90 | 1.47 | 0.6891 |  | 1.06 | 0.79 | 1.43 | 0.7051 | 67.4% |  | 1.36 | 0.89 | 2.09 | 0.1589 | 32.6% |
| C2 | 1.06 | 0.83 | 1.35 | 0.7871 |  | 0.96 | 0.69 | 1.33 | 0.8036 | 56.6% |  | 1.20 | 0.83 | 1.74 | 0.3341 | 43.4% |
| C3 | 1.11 | 0.86 | 1.45 | 0.6891 |  | 1.02 | 0.73 | 1.42 | 0.9145 | 63.5% |  | 1.30 | 0.84 | 2.01 | 0.2400 | 36.5% |
| C5-OH (C3-DC-M) | 1.27 | 1.00 | 1.62 | 0.5556 |  | 1.27 | 0.94 | 1.73 | 0.1225 | 63.4% |  | 1.27 | 0.85 | 1.90 | 0.2510 | 36.6% |
| C7-DC | 1.13 | 0.89 | 1.42 | 0.6891 |  | 1.24 | 0.91 | 1.68 | 0.1708 | 60.1% |  | 0.98 | 0.67 | 1.42 | 0.8971 | 39.9% |
| C9 | 1.02 | 0.79 | 1.31 | 0.9028 |  | 0.93 | 0.67 | 1.30 | 0.6702 | 57.0% |  | 1.15 | 0.78 | 1.69 | 0.4718 | 43.0% |
| C10 | 1.07 | 0.84 | 1.34 | 0.7785 |  | 1.11 | 0.85 | 1.45 | 0.4630 | 74.6% |  | 0.96 | 0.60 | 1.51 | 0.8452 | 25.4% |
| C10:2 | 1.06 | 0.83 | 1.34 | 0.7871 |  | 1.07 | 0.78 | 1.46 | 0.6882 | 58.1% |  | 1.04 | 0.72 | 1.51 | 0.8163 | 41.9% |
| C14:1 | 1.07 | 0.84 | 1.37 | 0.7781 |  | 1.01 | 0.73 | 1.39 | 0.9633 | 58.8% |  | 1.17 | 0.80 | 1.72 | 0.4197 | 41.2% |
| C14:2 | 1.06 | 0.83 | 1.35 | 0.7871 |  | 0.99 | 0.72 | 1.36 | 0.9554 | 58.6% |  | 1.15 | 0.79 | 1.69 | 0.4579 | 41.4% |
| C16 | 1.13 | 0.88 | 1.46 | 0.6891 |  | 0.86 | 0.62 | 1.19 | 0.3587 | 59.6% |  | 1.71 | 1.15 | 2.54 | 0.0079 | 40.4% |
| C18 | 1.39 | 1.09 | 1.79 | 0.3628 |  | 1.26 | 0.92 | 1.72 | 0.1552 | 62.5% |  | 1.66 | 1.10 | 2.48 | 0.0150 | 37.5% |
| C18:1 | 1.14 | 0.89 | 1.45 | 0.6891 |  | 0.96 | 0.70 | 1.31 | 0.8053 | 61.1% |  | 1.47 | 1.00 | 2.17 | 0.0525 | 38.9% |
| C18:2 | 1.02 | 0.80 | 1.29 | 0.8989 |  | 0.86 | 0.63 | 1.18 | 0.3393 | 56.9% |  | 1.28 | 0.89 | 1.84 | 0.1775 | 43.1% |
| **diacyl-Phosphatidylcholines** | |  |  |  |  |  |  |  |  |  |  |  |  |  |  |  |
| PC aa C28:1 | 0.89 | 0.68 | 1.16 | 0.6891 |  | 0.84 | 0.60 | 1.17 | 0.2951 | 65.8% |  | 0.99 | 0.62 | 1.56 | 0.9516 | 34.2% |
| PC aa C30:0 | 0.94 | 0.73 | 1.21 | 0.7871 |  | 0.84 | 0.60 | 1.18 | 0.3198 | 56.0% |  | 1.07 | 0.73 | 1.58 | 0.7201 | 44.0% |
| PC aa C32:0 | 0.97 | 0.76 | 1.25 | 0.8617 |  | 0.81 | 0.57 | 1.15 | 0.2336 | 51.3% |  | 1.18 | 0.82 | 1.68 | 0.3736 | 48.7% |
| PC aa C32:1 | 1.07 | 0.80 | 1.42 | 0.7871 |  | 0.71 | 0.42 | 1.19 | 0.1947 | 30.4% |  | 1.27 | 0.90 | 1.80 | 0.1661 | 69.6% |
| PC aa C32:2 | 0.85 | 0.63 | 1.14 | 0.6891 |  | 0.80 | 0.56 | 1.15 | 0.2288 | 67.3% |  | 0.95 | 0.57 | 1.60 | 0.8552 | 32.7% |
| PC aa C32:3 | 0.88 | 0.66 | 1.16 | 0.6891 |  | 0.70 | 0.50 | 0.98 | 0.0375 | 67.6% |  | 1.40 | 0.86 | 2.29 | 0.1745 | 32.4% |
| PC aa C34:1 | 1.09 | 0.85 | 1.39 | 0.7326 |  | 0.85 | 0.61 | 1.19 | 0.3376 | 56.3% |  | 1.49 | 1.02 | 2.18 | 0.0377 | 43.7% |
| PC aa C34:2 | 1.22 | 0.96 | 1.56 | 0.6891 |  | 1.01 | 0.74 | 1.37 | 0.9451 | 62.6% |  | 1.69 | 1.14 | 2.51 | 0.0096 | 37.4% |
| PC aa C34:3 | 0.97 | 0.73 | 1.27 | 0.8617 |  | 0.69 | 0.47 | 1.01 | 0.0539 | 51.8% |  | 1.40 | 0.94 | 2.08 | 0.0995 | 48.2% |
| PC aa C34:4 | 0.89 | 0.68 | 1.16 | 0.6891 |  | 0.79 | 0.57 | 1.10 | 0.1666 | 66.8% |  | 1.12 | 0.70 | 1.78 | 0.6291 | 33.2% |
| PC aa C36:0 | 0.88 | 0.67 | 1.14 | 0.6891 |  | 0.72 | 0.51 | 1.02 | 0.0678 | 57.6% |  | 1.13 | 0.76 | 1.70 | 0.5462 | 42.4% |
| PC aa C36:1 | 1.11 | 0.87 | 1.41 | 0.6891 |  | 1.01 | 0.75 | 1.37 | 0.9395 | 64.5% |  | 1.31 | 0.87 | 1.97 | 0.2015 | 35.5% |
| PC aa C36:2 | 1.19 | 0.93 | 1.53 | 0.6891 |  | 1.09 | 0.81 | 1.47 | 0.5771 | 68.1% |  | 1.44 | 0.93 | 2.24 | 0.1015 | 31.9% |
| PC aa C36:3 | 1.26 | 0.99 | 1.60 | 0.5556 |  | 1.10 | 0.82 | 1.48 | 0.5332 | 65.7% |  | 1.65 | 1.09 | 2.48 | 0.0170 | 34.3% |
| PC aa C36:4 | 1.14 | 0.89 | 1.46 | 0.6891 |  | 0.98 | 0.71 | 1.35 | 0.9021 | 59.4% |  | 1.42 | 0.96 | 2.08 | 0.0768 | 40.6% |
| PC aa C36:5 | 0.89 | 0.68 | 1.15 | 0.6891 |  | 0.83 | 0.59 | 1.17 | 0.2887 | 56.7% |  | 0.97 | 0.65 | 1.45 | 0.8916 | 43.3% |
| PC aa C36:6 | 0.80 | 0.60 | 1.06 | 0.6891 |  | 0.69 | 0.48 | 1.00 | 0.0474 | 59.3% |  | 1.00 | 0.64 | 1.55 | 0.9937 | 40.7% |
| PC aa C38:0 | 0.86 | 0.66 | 1.11 | 0.6891 |  | 0.71 | 0.51 | 1.00 | 0.0486 | 58.9% |  | 1.12 | 0.75 | 1.69 | 0.5797 | 41.1% |
| PC aa C38:1 | 0.93 | 0.72 | 1.19 | 0.7781 |  | 0.78 | 0.56 | 1.08 | 0.1290 | 58.0% |  | 1.20 | 0.81 | 1.76 | 0.3663 | 42.0% |
| PC aa C38:3 | 1.33 | 1.03 | 1.70 | 0.4518 |  | 1.24 | 0.91 | 1.68 | 0.1697 | 65.7% |  | 1.51 | 0.99 | 2.30 | 0.0585 | 34.3% |
| PC aa C38:4 | 1.16 | 0.90 | 1.49 | 0.6891 |  | 1.09 | 0.79 | 1.49 | 0.6161 | 63.1% |  | 1.30 | 0.86 | 1.98 | 0.2135 | 36.9% |
| PC aa C38:5 | 0.97 | 0.75 | 1.25 | 0.8617 |  | 0.86 | 0.61 | 1.20 | 0.3593 | 57.5% |  | 1.15 | 0.78 | 1.70 | 0.4760 | 42.5% |
| PC aa C38:6 | 0.95 | 0.74 | 1.22 | 0.8024 |  | 0.77 | 0.55 | 1.07 | 0.1233 | 54.3% |  | 1.23 | 0.85 | 1.77 | 0.2718 | 45.7% |
| PC aa C40:2 | 0.69 | 0.52 | 0.91 | 0.3628 |  | 0.63 | 0.44 | 0.90 | 0.0102 | 63.7% |  | 0.80 | 0.50 | 1.27 | 0.3412 | 36.3% |
| PC aa C40:3 | 0.82 | 0.63 | 1.06 | 0.6891 |  | 0.72 | 0.51 | 1.02 | 0.0615 | 59.6% |  | 0.98 | 0.65 | 1.48 | 0.9147 | 40.4% |
| PC aa C40:4 | 1.13 | 0.88 | 1.45 | 0.6891 |  | 1.03 | 0.75 | 1.40 | 0.8755 | 62.4% |  | 1.33 | 0.89 | 1.99 | 0.1616 | 37.6% |
| PC aa C40:5 | 1.06 | 0.82 | 1.37 | 0.7871 |  | 0.90 | 0.64 | 1.26 | 0.5371 | 55.3% |  | 1.31 | 0.90 | 1.91 | 0.1651 | 44.7% |
| PC aa C40:6 | 1.03 | 0.80 | 1.33 | 0.8617 |  | 0.87 | 0.62 | 1.21 | 0.4087 | 56.6% |  | 1.29 | 0.88 | 1.89 | 0.1871 | 43.4% |
| PC aa C42:0 | 0.79 | 0.61 | 1.04 | 0.6891 |  | 0.68 | 0.48 | 0.96 | 0.0267 | 60.9% |  | 1.01 | 0.66 | 1.54 | 0.9702 | 39.1% |
| PC aa C42:1 | 0.73 | 0.55 | 0.96 | 0.4518 |  | 0.56 | 0.39 | 0.81 | 0.0017 | 57.6% |  | 1.04 | 0.68 | 1.59 | 0.8470 | 42.4% |
| PC aa C42:2 | 0.89 | 0.69 | 1.14 | 0.6891 |  | 0.68 | 0.48 | 0.97 | 0.0319 | 51.3% |  | 1.17 | 0.82 | 1.68 | 0.3875 | 48.7% |
| PC aa C42:4 | 0.99 | 0.77 | 1.27 | 0.9452 |  | 0.85 | 0.62 | 1.16 | 0.3008 | 61.9% |  | 1.28 | 0.85 | 1.92 | 0.2324 | 38.1% |
| PC aa C42:5 | 0.91 | 0.70 | 1.18 | 0.7326 |  | 0.76 | 0.54 | 1.07 | 0.1138 | 56.0% |  | 1.16 | 0.78 | 1.71 | 0.4581 | 44.0% |
| PC aa C42:6 | 0.81 | 0.62 | 1.06 | 0.6891 |  | 0.62 | 0.43 | 0.89 | 0.0098 | 55.8% |  | 1.14 | 0.76 | 1.71 | 0.5352 | 44.2% |
| **acyl-alkyl-Phosphatidylcholines** | |  |  |  |  |  |  |  |  |  |  |  |  |  |  |  |
| PC ae C30:0 | 0.83 | 0.63 | 1.10 | 0.6891 |  | 0.73 | 0.51 | 1.05 | 0.0913 | 59.2% |  | 1.01 | 0.65 | 1.57 | 0.9609 | 40.8% |
| PC ae C30:2 | 0.85 | 0.64 | 1.13 | 0.6891 |  | 0.85 | 0.59 | 1.22 | 0.3782 | 63.3% |  | 0.85 | 0.53 | 1.37 | 0.5063 | 36.7% |
| PC ae C32:1 | 0.76 | 0.57 | 1.00 | 0.5556 |  | 0.61 | 0.42 | 0.88 | 0.0087 | 60.4% |  | 1.04 | 0.66 | 1.64 | 0.8618 | 39.6% |
| PC ae C32:2 | 0.71 | 0.53 | 0.95 | 0.4518 |  | 0.62 | 0.43 | 0.88 | 0.0080 | 66.4% |  | 0.93 | 0.56 | 1.55 | 0.7872 | 33.6% |
| PC ae C34:0 | 0.88 | 0.67 | 1.15 | 0.6891 |  | 0.80 | 0.57 | 1.12 | 0.1934 | 62.9% |  | 1.03 | 0.67 | 1.59 | 0.8989 | 37.1% |
| PC ae C34:1 | 0.95 | 0.73 | 1.24 | 0.8195 |  | 0.80 | 0.57 | 1.12 | 0.1991 | 62.7% |  | 1.27 | 0.82 | 1.96 | 0.2779 | 37.3% |
| PC ae C34:2 | 0.92 | 0.70 | 1.22 | 0.7781 |  | 0.88 | 0.63 | 1.24 | 0.4667 | 65.9% |  | 1.02 | 0.63 | 1.63 | 0.9508 | 34.1% |
| PC ae C34:3 | 0.85 | 0.64 | 1.13 | 0.6891 |  | 0.82 | 0.59 | 1.14 | 0.2380 | 70.5% |  | 0.93 | 0.56 | 1.56 | 0.7932 | 29.5% |
| PC ae C36:0 | 0.84 | 0.64 | 1.10 | 0.6891 |  | 0.70 | 0.48 | 1.03 | 0.0694 | 49.5% |  | 1.00 | 0.68 | 1.45 | 0.9885 | 50.5% |
| PC ae C36:1 | 0.92 | 0.70 | 1.19 | 0.7326 |  | 0.81 | 0.59 | 1.13 | 0.2103 | 66.4% |  | 1.16 | 0.73 | 1.84 | 0.5229 | 33.6% |
| PC ae C36:2 | 0.96 | 0.72 | 1.28 | 0.8617 |  | 0.85 | 0.60 | 1.20 | 0.3641 | 68.0% |  | 1.24 | 0.75 | 2.05 | 0.4078 | 32.0% |
| PC ae C36:3 | 1.00 | 0.77 | 1.31 | 0.9899 |  | 0.94 | 0.68 | 1.30 | 0.6911 | 67.0% |  | 1.15 | 0.72 | 1.83 | 0.5572 | 33.0% |
| PC ae C36:4 | 1.06 | 0.83 | 1.36 | 0.7871 |  | 1.01 | 0.74 | 1.38 | 0.9631 | 62.7% |  | 1.15 | 0.77 | 1.73 | 0.4959 | 37.3% |
| PC ae C36:5 | 0.91 | 0.70 | 1.18 | 0.7326 |  | 0.87 | 0.63 | 1.20 | 0.3818 | 64.3% |  | 1.01 | 0.65 | 1.56 | 0.9775 | 35.7% |
| PC ae C38:0 | 0.83 | 0.63 | 1.08 | 0.6891 |  | 0.67 | 0.47 | 0.96 | 0.0272 | 56.6% |  | 1.09 | 0.73 | 1.63 | 0.6831 | 43.4% |
| PC ae C38:1 | 0.82 | 0.64 | 1.07 | 0.6891 |  | 0.80 | 0.58 | 1.11 | 0.1755 | 64.9% |  | 0.87 | 0.56 | 1.34 | 0.5204 | 35.1% |
| PC ae C38:2 | 0.86 | 0.65 | 1.13 | 0.6891 |  | 0.69 | 0.49 | 0.98 | 0.0367 | 64.2% |  | 1.27 | 0.80 | 2.02 | 0.3082 | 35.8% |
| PC ae C38:3 | 1.09 | 0.84 | 1.42 | 0.7326 |  | 0.99 | 0.72 | 1.37 | 0.9719 | 66.7% |  | 1.33 | 0.84 | 2.10 | 0.2250 | 33.3% |
| PC ae C38:4 | 1.16 | 0.90 | 1.50 | 0.6891 |  | 1.07 | 0.78 | 1.48 | 0.6783 | 61.9% |  | 1.33 | 0.88 | 2.01 | 0.1732 | 38.1% |
| PC ae C38:5 | 1.01 | 0.79 | 1.31 | 0.9253 |  | 0.92 | 0.67 | 1.27 | 0.6115 | 61.4% |  | 1.19 | 0.79 | 1.78 | 0.4102 | 38.6% |
| PC ae C38:6 | 0.85 | 0.66 | 1.10 | 0.6891 |  | 0.77 | 0.56 | 1.07 | 0.1201 | 62.6% |  | 0.99 | 0.65 | 1.51 | 0.9714 | 37.4% |
| PC ae C40:1 | 0.91 | 0.71 | 1.17 | 0.7159 |  | 0.75 | 0.54 | 1.05 | 0.0905 | 57.3% |  | 1.18 | 0.80 | 1.73 | 0.4056 | 42.7% |
| PC ae C40:2 | 0.90 | 0.69 | 1.17 | 0.6891 |  | 0.84 | 0.61 | 1.17 | 0.3019 | 66.4% |  | 1.02 | 0.65 | 1.60 | 0.9428 | 33.6% |
| PC ae C40:3 | 0.93 | 0.71 | 1.22 | 0.7871 |  | 0.82 | 0.59 | 1.16 | 0.2628 | 63.9% |  | 1.17 | 0.74 | 1.83 | 0.5028 | 36.1% |
| PC ae C40:4 | 1.09 | 0.84 | 1.42 | 0.7326 |  | 0.95 | 0.68 | 1.32 | 0.7409 | 61.1% |  | 1.37 | 0.90 | 2.09 | 0.1409 | 38.9% |
| PC ae C40:5 | 0.96 | 0.73 | 1.28 | 0.8617 |  | 0.81 | 0.56 | 1.18 | 0.2758 | 57.3% |  | 1.21 | 0.79 | 1.86 | 0.3811 | 42.7% |
| PC ae C40:6 | 0.84 | 0.64 | 1.11 | 0.6891 |  | 0.67 | 0.48 | 0.95 | 0.0250 | 62.1% |  | 1.22 | 0.79 | 1.90 | 0.3706 | 37.9% |
| PC ae C42:1 | 0.89 | 0.69 | 1.15 | 0.6891 |  | 0.79 | 0.57 | 1.11 | 0.1739 | 61.4% |  | 1.07 | 0.70 | 1.62 | 0.7654 | 38.6% |
| PC ae C42:2 | 0.86 | 0.66 | 1.13 | 0.6891 |  | 0.72 | 0.50 | 1.02 | 0.0640 | 59.1% |  | 1.13 | 0.74 | 1.72 | 0.5781 | 40.9% |
| PC ae C42:3 | 0.89 | 0.69 | 1.16 | 0.6891 |  | 0.74 | 0.53 | 1.04 | 0.0856 | 57.9% |  | 1.15 | 0.77 | 1.72 | 0.4846 | 42.1% |
| PC ae C42:4 | 1.12 | 0.87 | 1.45 | 0.6891 |  | 1.02 | 0.73 | 1.43 | 0.8936 | 59.3% |  | 1.29 | 0.86 | 1.93 | 0.2210 | 40.7% |
| PC ae C42:5 | 0.89 | 0.69 | 1.16 | 0.6891 |  | 0.76 | 0.55 | 1.06 | 0.1115 | 62.2% |  | 1.16 | 0.76 | 1.78 | 0.4873 | 37.8% |
| PC ae C44:3 | 0.81 | 0.62 | 1.06 | 0.6891 |  | 0.60 | 0.42 | 0.87 | 0.0065 | 54.7% |  | 1.15 | 0.77 | 1.72 | 0.4927 | 45.3% |
| PC ae C44:4 | 1.16 | 0.90 | 1.49 | 0.6891 |  | 1.06 | 0.76 | 1.47 | 0.7508 | 60.1% |  | 1.33 | 0.89 | 2.00 | 0.1681 | 39.9% |
| PC ae C44:5 | 0.95 | 0.74 | 1.22 | 0.8024 |  | 0.79 | 0.57 | 1.10 | 0.1619 | 58.7% |  | 1.24 | 0.84 | 1.82 | 0.2884 | 41.3% |
| PC ae C44:6 | 0.88 | 0.69 | 1.14 | 0.6891 |  | 0.70 | 0.50 | 0.98 | 0.0351 | 56.5% |  | 1.20 | 0.82 | 1.77 | 0.3397 | 43.5% |
| **lyso-Phosphatidylcholines** | |  |  |  |  |  |  |  |  |  |  |  |  |  |  |  |
| lysoPC a C14:0 | 0.97 | 0.74 | 1.26 | 0.8617 |  | 0.86 | 0.60 | 1.25 | 0.4347 | 53.2% |  | 1.10 | 0.74 | 1.63 | 0.6442 | 46.8% |
| lysoPC a C16:0 | 0.89 | 0.68 | 1.16 | 0.6891 |  | 0.77 | 0.54 | 1.10 | 0.1510 | 55.9% |  | 1.06 | 0.71 | 1.59 | 0.7720 | 44.1% |
| lysoPC a C16:1 | 0.90 | 0.68 | 1.19 | 0.7158 |  | 0.65 | 0.41 | 1.02 | 0.0589 | 39.3% |  | 1.11 | 0.77 | 1.59 | 0.5738 | 60.7% |
| lysoPC a C17:0 | 0.74 | 0.55 | 0.99 | 0.5556 |  | 0.67 | 0.46 | 0.97 | 0.0347 | 62.9% |  | 0.86 | 0.53 | 1.40 | 0.5491 | 37.1% |
| lysoPC a C18:0 | 0.83 | 0.63 | 1.09 | 0.6891 |  | 0.82 | 0.58 | 1.16 | 0.2578 | 64.4% |  | 0.84 | 0.53 | 1.34 | 0.4736 | 35.6% |
| lysoPC a C18:1 | 0.82 | 0.62 | 1.09 | 0.6891 |  | 0.73 | 0.50 | 1.06 | 0.0951 | 56.4% |  | 0.96 | 0.62 | 1.47 | 0.8416 | 43.6% |
| lysoPC a C18:2 | 0.87 | 0.65 | 1.16 | 0.6891 |  | 0.89 | 0.62 | 1.28 | 0.5190 | 63.7% |  | 0.85 | 0.52 | 1.37 | 0.4956 | 36.3% |
| lysoPC a C20:3 | 1.05 | 0.82 | 1.35 | 0.8024 |  | 1.03 | 0.75 | 1.43 | 0.8508 | 60.7% |  | 1.08 | 0.73 | 1.62 | 0.6944 | 39.3% |
| lysoPC a C20:4 | 0.83 | 0.64 | 1.09 | 0.6891 |  | 0.82 | 0.58 | 1.16 | 0.2619 | 58.6% |  | 0.85 | 0.56 | 1.29 | 0.4536 | 41.4% |
| lysoPC a C28:1 | 0.74 | 0.54 | 1.00 | 0.5556 |  | 0.72 | 0.49 | 1.06 | 0.0989 | 61.4% |  | 0.77 | 0.47 | 1.26 | 0.2990 | 38.6% |
| **Sphingomyelins** |  |  |  |  |  |  |  |  |  |  |  |  |  |  |  |  |
| SM C16:0 | 1.22 | 0.95 | 1.57 | 0.6891 |  | 1.08 | 0.79 | 1.49 | 0.6181 | 63.9% |  | 1.50 | 0.99 | 2.28 | 0.0561 | 36.1% |
| SM C16:1 | 1.29 | 0.99 | 1.67 | 0.5556 |  | 1.14 | 0.84 | 1.55 | 0.4097 | 72.4% |  | 1.77 | 1.08 | 2.92 | 0.0244 | 27.6% |
| SM C18:0 | 1.35 | 1.06 | 1.73 | 0.4518 |  | 1.37 | 1.01 | 1.86 | 0.0448 | 64.8% |  | 1.32 | 0.87 | 2.00 | 0.1932 | 35.2% |
| SM C18:1 | 1.41 | 1.10 | 1.80 | 0.3628 |  | 1.36 | 1.02 | 1.81 | 0.0352 | 73.4% |  | 1.55 | 0.96 | 2.49 | 0.0733 | 26.6% |
| SM C20:2 | 0.92 | 0.69 | 1.22 | 0.7693 |  | 0.82 | 0.57 | 1.17 | 0.2664 | 63.6% |  | 1.12 | 0.70 | 1.80 | 0.6276 | 36.4% |
| SM C24:0 | 1.18 | 0.92 | 1.51 | 0.6891 |  | 1.22 | 0.90 | 1.65 | 0.1928 | 66.3% |  | 1.11 | 0.73 | 1.69 | 0.6361 | 33.7% |
| SM C24:1 | 1.16 | 0.91 | 1.48 | 0.6891 |  | 1.06 | 0.78 | 1.44 | 0.7126 | 62.1% |  | 1.34 | 0.91 | 1.99 | 0.1396 | 37.9% |
| SM C26:1 | 1.13 | 0.89 | 1.42 | 0.6891 |  | 1.13 | 0.84 | 1.52 | 0.4110 | 62.3% |  | 1.12 | 0.77 | 1.63 | 0.5631 | 37.7% |
| SM (OH) C14:1 | 1.04 | 0.79 | 1.36 | 0.8617 |  | 1.05 | 0.76 | 1.46 | 0.7555 | 70.5% |  | 1.00 | 0.60 | 1.65 | 0.9894 | 29.5% |
| SM (OH) C16:1 | 1.13 | 0.87 | 1.47 | 0.6891 |  | 1.14 | 0.83 | 1.55 | 0.4246 | 69.8% |  | 1.13 | 0.70 | 1.82 | 0.6123 | 30.2% |
| SM (OH) C22:1 | 1.14 | 0.88 | 1.48 | 0.6891 |  | 1.17 | 0.86 | 1.59 | 0.3069 | 72.1% |  | 1.05 | 0.64 | 1.72 | 0.8444 | 27.9% |
| SM (OH) C22:2 | 1.12 | 0.86 | 1.46 | 0.6891 |  | 1.15 | 0.85 | 1.56 | 0.3536 | 77.0% |  | 1.03 | 0.59 | 1.78 | 0.9273 | 23.0% |
| SM (OH) C24:1 | 0.95 | 0.74 | 1.23 | 0.8091 |  | 1.01 | 0.74 | 1.37 | 0.9655 | 70.8% |  | 0.83 | 0.52 | 1.34 | 0.4465 | 29.2% |

a, acyl; AC, acylcarnitines; e, alkyl; LCL, lower 95% confidence limit; OR, odds ratio; PC, phosphatidylcholines; SM, sphingomyelin; UCL, upper 95% confidence limit
