# Supplementary material for: Specific Metabolic Markers Are Associated with Future Waist-Gaining Phenotype in Women
Source: PLoS One. 2016 Jun 20;11(6):e0157733. doi: 10.1371/journal.pone.0157733 (PMC4920591; doi:10.1371/journal.pone.0157733)
Supplement: S6 Table — (DOCX) [file pone.0157733.s006.docx]

Table S6 Association of metabolites with hip-gaining phenotype in men in the combined fixed-effect meta-analysis and by specific study

|  | **overall** | | | |  | **EPIC-Potsdam** | | | | |  | **KORA** | | | | |
| --- | --- | --- | --- | --- | --- | --- | --- | --- | --- | --- | --- | --- | --- | --- | --- | --- |
| **Amino Acids** | **OR** | **LCL** | **UCL** | **FDR p** |  | **OR** | **LCL** | **UCL** | **uncorrected  p value** | **meta weight** |  | **OR** | **LCL** | **UCL** | **uncorrected  p value** | **meta weight** |
| Arg | 0.86 | 0.66 | 1.11 | 0.9943 |  | 0.89 | 0.65 | 1.22 | 0.4737 | 67.5% |  | 0.79 | 0.50 | 1.25 | 0.3084 | 32.5% |
| Gln | 0.94 | 0.73 | 1.22 | 0.9943 |  | 0.92 | 0.67 | 1.26 | 0.6045 | 66.6% |  | 0.98 | 0.63 | 1.53 | 0.9324 | 33.4% |
| Gly | 0.87 | 0.65 | 1.16 | 0.9943 |  | 1.02 | 0.74 | 1.40 | 0.9244 | 80.2% |  | 0.47 | 0.24 | 0.89 | 0.0217 | 19.8% |
| His | 0.84 | 0.65 | 1.09 | 0.9943 |  | 0.85 | 0.61 | 1.17 | 0.3045 | 64.5% |  | 0.84 | 0.54 | 1.29 | 0.4257 | 35.5% |
| Met | 0.79 | 0.61 | 1.03 | 0.9943 |  | 0.85 | 0.61 | 1.19 | 0.3411 | 62.6% |  | 0.71 | 0.46 | 1.09 | 0.1161 | 37.4% |
| Orn | 0.73 | 0.54 | 0.98 | 0.9943 |  | 0.79 | 0.56 | 1.11 | 0.1785 | 72.3% |  | 0.59 | 0.34 | 1.03 | 0.0649 | 27.7% |
| Phe | 0.88 | 0.68 | 1.13 | 0.9943 |  | 1.01 | 0.75 | 1.36 | 0.9442 | 75.1% |  | 0.57 | 0.34 | 0.96 | 0.0331 | 24.9% |
| Pro | 1.04 | 0.82 | 1.32 | 0.9943 |  | 1.05 | 0.78 | 1.41 | 0.7532 | 66.0% |  | 1.01 | 0.67 | 1.53 | 0.9480 | 34.0% |
| Ser | 0.75 | 0.56 | 1.00 | 0.9943 |  | 0.72 | 0.50 | 1.03 | 0.0732 | 65.8% |  | 0.81 | 0.49 | 1.33 | 0.4053 | 34.2% |
| Thr | 0.83 | 0.64 | 1.08 | 0.9943 |  | 0.76 | 0.54 | 1.07 | 0.1190 | 60.0% |  | 0.93 | 0.61 | 1.41 | 0.7376 | 40.0% |
| Trp | 0.85 | 0.66 | 1.09 | 0.9943 |  | 0.82 | 0.60 | 1.11 | 0.1981 | 64.5% |  | 0.92 | 0.61 | 1.40 | 0.7092 | 35.5% |
| Tyr | 0.87 | 0.67 | 1.13 | 0.9943 |  | 0.96 | 0.69 | 1.33 | 0.8022 | 64.9% |  | 0.73 | 0.47 | 1.13 | 0.1569 | 35.1% |
| Val | 0.83 | 0.63 | 1.09 | 0.9943 |  | 0.96 | 0.69 | 1.33 | 0.7936 | 69.9% |  | 0.59 | 0.36 | 0.98 | 0.0414 | 30.1% |
| **Hexose** |  |  |  |  |  |  |  |  |  |  |  |  |  |  |  |  |
| H1 | 1.13 | 0.85 | 1.49 | 0.9943 |  | 1.09 | 0.78 | 1.51 | 0.6262 | 72.8% |  | 1.26 | 0.74 | 2.15 | 0.4035 | 27.2% |
| **Acylcarnitines** |  |  |  |  |  |  |  |  |  |  |  |  |  |  |  |  |
| C0 | 1.06 | 0.82 | 1.36 | 0.9943 |  | 1.04 | 0.76 | 1.41 | 0.8165 | 67.3% |  | 1.11 | 0.71 | 1.73 | 0.6436 | 32.7% |
| C2 | 1.04 | 0.83 | 1.29 | 0.9943 |  | 1.01 | 0.78 | 1.30 | 0.9455 | 75.8% |  | 1.13 | 0.72 | 1.77 | 0.5830 | 24.2% |
| C3 | 1.09 | 0.85 | 1.40 | 0.9943 |  | 1.23 | 0.92 | 1.65 | 0.1680 | 70.6% |  | 0.82 | 0.52 | 1.29 | 0.3796 | 29.4% |
| C5-OH (C3-DC-M) | 1.05 | 0.83 | 1.32 | 0.9943 |  | 1.07 | 0.81 | 1.42 | 0.6369 | 66.2% |  | 1.00 | 0.67 | 1.48 | 0.9842 | 33.8% |
| C7-DC | 1.04 | 0.82 | 1.31 | 0.9943 |  | 1.05 | 0.78 | 1.40 | 0.7591 | 65.9% |  | 1.02 | 0.68 | 1.53 | 0.9332 | 34.1% |
| C9 | 1.05 | 0.82 | 1.33 | 0.9943 |  | 1.03 | 0.77 | 1.39 | 0.8400 | 66.1% |  | 1.08 | 0.71 | 1.64 | 0.7169 | 33.9% |
| C10 | 0.95 | 0.75 | 1.22 | 0.9943 |  | 0.91 | 0.67 | 1.22 | 0.5173 | 66.6% |  | 1.05 | 0.69 | 1.60 | 0.8109 | 33.4% |
| C10:2 | 0.91 | 0.71 | 1.17 | 0.9943 |  | 0.83 | 0.61 | 1.13 | 0.2303 | 64.1% |  | 1.09 | 0.72 | 1.65 | 0.6842 | 35.9% |
| C14:1 | 1.02 | 0.80 | 1.29 | 0.9943 |  | 0.99 | 0.73 | 1.34 | 0.9301 | 60.5% |  | 1.06 | 0.73 | 1.55 | 0.7476 | 39.5% |
| C14:2 | 0.98 | 0.78 | 1.23 | 0.9943 |  | 0.86 | 0.63 | 1.17 | 0.3361 | 56.7% |  | 1.15 | 0.81 | 1.64 | 0.4246 | 43.3% |
| C16 | 1.08 | 0.84 | 1.38 | 0.9943 |  | 1.07 | 0.80 | 1.44 | 0.6552 | 69.5% |  | 1.11 | 0.71 | 1.73 | 0.6530 | 30.5% |
| C18 | 1.33 | 1.04 | 1.69 | 0.9943 |  | 1.50 | 1.12 | 2.00 | 0.0063 | 69.5% |  | 1.02 | 0.66 | 1.57 | 0.9448 | 30.5% |
| C18:1 | 1.30 | 1.04 | 1.63 | 0.9943 |  | 1.40 | 1.06 | 1.85 | 0.0167 | 65.8% |  | 1.12 | 0.76 | 1.65 | 0.5586 | 34.2% |
| C18:2 | 1.16 | 0.92 | 1.47 | 0.9943 |  | 1.26 | 0.95 | 1.66 | 0.1072 | 69.9% |  | 0.97 | 0.63 | 1.48 | 0.8728 | 30.1% |
| **diacyl-Phosphatidylcholines** | |  |  |  |  |  |  |  |  |  |  |  |  |  |  |  |
| PC aa C28:1 | 1.17 | 0.91 | 1.50 | 0.9943 |  | 1.35 | 1.01 | 1.78 | 0.0395 | 76.4% |  | 0.75 | 0.45 | 1.24 | 0.2555 | 23.6% |
| PC aa C30:0 | 1.01 | 0.79 | 1.29 | 0.9943 |  | 1.14 | 0.86 | 1.51 | 0.3545 | 76.8% |  | 0.68 | 0.41 | 1.13 | 0.1352 | 23.2% |
| PC aa C32:0 | 1.00 | 0.78 | 1.28 | 0.9958 |  | 1.03 | 0.75 | 1.41 | 0.8500 | 62.9% |  | 0.95 | 0.63 | 1.43 | 0.7973 | 37.1% |
| PC aa C32:1 | 0.94 | 0.73 | 1.21 | 0.9943 |  | 1.01 | 0.72 | 1.42 | 0.9564 | 57.3% |  | 0.85 | 0.57 | 1.26 | 0.4121 | 42.7% |
| PC aa C32:2 | 1.10 | 0.86 | 1.40 | 0.9943 |  | 1.20 | 0.92 | 1.58 | 0.1814 | 81.9% |  | 0.72 | 0.41 | 1.28 | 0.2615 | 18.1% |
| PC aa C32:3 | 0.98 | 0.75 | 1.29 | 0.9943 |  | 0.96 | 0.70 | 1.32 | 0.8040 | 72.7% |  | 1.04 | 0.62 | 1.75 | 0.8913 | 27.3% |
| PC aa C34:1 | 0.95 | 0.74 | 1.22 | 0.9943 |  | 0.94 | 0.69 | 1.28 | 0.7102 | 64.2% |  | 0.96 | 0.63 | 1.45 | 0.8454 | 35.8% |
| PC aa C34:2 | 1.04 | 0.81 | 1.34 | 0.9943 |  | 0.98 | 0.71 | 1.34 | 0.8846 | 63.1% |  | 1.17 | 0.77 | 1.76 | 0.4578 | 36.9% |
| PC aa C34:3 | 1.00 | 0.77 | 1.30 | 0.9943 |  | 1.06 | 0.78 | 1.45 | 0.7123 | 69.2% |  | 0.89 | 0.55 | 1.41 | 0.6113 | 30.8% |
| PC aa C34:4 | 1.04 | 0.80 | 1.35 | 0.9943 |  | 1.18 | 0.87 | 1.59 | 0.2923 | 73.8% |  | 0.73 | 0.44 | 1.22 | 0.2328 | 26.2% |
| PC aa C36:0 | 0.97 | 0.75 | 1.25 | 0.9943 |  | 0.96 | 0.70 | 1.33 | 0.8218 | 66.0% |  | 0.98 | 0.63 | 1.52 | 0.9198 | 34.0% |
| PC aa C36:1 | 0.92 | 0.71 | 1.19 | 0.9943 |  | 0.96 | 0.70 | 1.30 | 0.7764 | 69.7% |  | 0.85 | 0.53 | 1.36 | 0.4934 | 30.3% |
| PC aa C36:2 | 1.03 | 0.80 | 1.33 | 0.9943 |  | 0.95 | 0.70 | 1.31 | 0.7649 | 65.9% |  | 1.20 | 0.77 | 1.85 | 0.4252 | 34.1% |
| PC aa C36:3 | 1.09 | 0.85 | 1.40 | 0.9943 |  | 1.07 | 0.79 | 1.46 | 0.6617 | 63.7% |  | 1.12 | 0.75 | 1.69 | 0.5754 | 36.3% |
| PC aa C36:4 | 1.06 | 0.84 | 1.34 | 0.9943 |  | 1.11 | 0.83 | 1.50 | 0.4864 | 61.8% |  | 0.98 | 0.67 | 1.44 | 0.9346 | 38.2% |
| PC aa C36:5 | 0.90 | 0.68 | 1.18 | 0.9943 |  | 1.00 | 0.73 | 1.37 | 0.9996 | 73.8% |  | 0.66 | 0.39 | 1.13 | 0.1326 | 26.2% |
| PC aa C36:6 | 0.97 | 0.74 | 1.27 | 0.9943 |  | 1.11 | 0.81 | 1.51 | 0.5226 | 75.2% |  | 0.66 | 0.38 | 1.13 | 0.1290 | 24.8% |
| PC aa C38:0 | 0.87 | 0.66 | 1.15 | 0.9943 |  | 0.85 | 0.61 | 1.20 | 0.3630 | 66.2% |  | 0.90 | 0.56 | 1.45 | 0.6674 | 33.8% |
| PC aa C38:1 | 0.74 | 0.56 | 0.98 | 0.9943 |  | 0.67 | 0.47 | 0.97 | 0.0326 | 59.9% |  | 0.86 | 0.55 | 1.34 | 0.5098 | 40.1% |
| PC aa C38:3 | 1.07 | 0.82 | 1.38 | 0.9943 |  | 1.09 | 0.79 | 1.52 | 0.5946 | 62.0% |  | 1.03 | 0.68 | 1.56 | 0.9017 | 38.0% |
| PC aa C38:4 | 1.05 | 0.83 | 1.33 | 0.9943 |  | 1.09 | 0.81 | 1.47 | 0.5726 | 63.4% |  | 0.99 | 0.67 | 1.47 | 0.9759 | 36.6% |
| PC aa C38:5 | 0.99 | 0.78 | 1.26 | 0.9943 |  | 1.05 | 0.78 | 1.42 | 0.7479 | 64.1% |  | 0.89 | 0.60 | 1.33 | 0.5736 | 35.9% |
| PC aa C38:6 | 0.97 | 0.76 | 1.24 | 0.9943 |  | 0.98 | 0.72 | 1.34 | 0.8940 | 62.9% |  | 0.96 | 0.64 | 1.44 | 0.8290 | 37.1% |
| PC aa C40:2 | 0.78 | 0.59 | 1.04 | 0.9943 |  | 0.72 | 0.50 | 1.03 | 0.0751 | 61.7% |  | 0.89 | 0.56 | 1.40 | 0.6048 | 38.3% |
| PC aa C40:3 | 0.85 | 0.65 | 1.13 | 0.9943 |  | 0.70 | 0.48 | 1.04 | 0.0739 | 51.5% |  | 1.05 | 0.71 | 1.57 | 0.8009 | 48.5% |
| PC aa C40:4 | 1.10 | 0.88 | 1.38 | 0.9943 |  | 1.07 | 0.79 | 1.44 | 0.6698 | 58.3% |  | 1.16 | 0.82 | 1.65 | 0.4098 | 41.7% |
| PC aa C40:5 | 1.06 | 0.84 | 1.34 | 0.9943 |  | 1.11 | 0.83 | 1.49 | 0.4919 | 63.1% |  | 0.99 | 0.68 | 1.46 | 0.9752 | 36.9% |
| PC aa C40:6 | 0.96 | 0.75 | 1.24 | 0.9943 |  | 0.99 | 0.72 | 1.36 | 0.9374 | 62.6% |  | 0.92 | 0.61 | 1.40 | 0.7005 | 37.4% |
| PC aa C42:0 | 1.05 | 0.80 | 1.36 | 0.9943 |  | 1.10 | 0.80 | 1.51 | 0.5509 | 70.1% |  | 0.93 | 0.57 | 1.51 | 0.7653 | 29.9% |
| PC aa C42:1 | 1.16 | 0.90 | 1.49 | 0.9943 |  | 1.26 | 0.94 | 1.70 | 0.1211 | 73.5% |  | 0.91 | 0.56 | 1.49 | 0.7031 | 26.5% |
| PC aa C42:2 | 1.07 | 0.83 | 1.37 | 0.9943 |  | 1.07 | 0.79 | 1.45 | 0.6633 | 65.3% |  | 1.06 | 0.70 | 1.61 | 0.7900 | 34.7% |
| PC aa C42:4 | 0.84 | 0.65 | 1.10 | 0.9943 |  | 0.90 | 0.66 | 1.23 | 0.5118 | 68.8% |  | 0.73 | 0.46 | 1.17 | 0.1917 | 31.2% |
| PC aa C42:5 | 0.86 | 0.66 | 1.13 | 0.9943 |  | 0.77 | 0.54 | 1.09 | 0.1438 | 57.8% |  | 1.01 | 0.67 | 1.53 | 0.9627 | 42.2% |
| PC aa C42:6 | 1.08 | 0.84 | 1.38 | 0.9943 |  | 1.14 | 0.83 | 1.55 | 0.4230 | 62.6% |  | 0.99 | 0.66 | 1.48 | 0.9607 | 37.4% |
| **acyl-alkyl-Phosphatidylcholines** | | |  |  |  |  |  |  |  |  |  |  |  |  |  |  |
| PC ae C30:0 | 1.05 | 0.82 | 1.35 | 0.9943 |  | 1.18 | 0.89 | 1.56 | 0.2531 | 81.7% |  | 0.64 | 0.35 | 1.15 | 0.1339 | 18.3% |
| PC ae C30:2 | 1.23 | 0.97 | 1.55 | 0.9943 |  | 1.27 | 0.96 | 1.68 | 0.0914 | 71.2% |  | 1.12 | 0.72 | 1.74 | 0.6040 | 28.8% |
| PC ae C32:1 | 0.91 | 0.71 | 1.19 | 0.9943 |  | 0.97 | 0.72 | 1.32 | 0.8670 | 72.4% |  | 0.78 | 0.47 | 1.27 | 0.3131 | 27.6% |
| PC ae C32:2 | 0.88 | 0.67 | 1.15 | 0.9943 |  | 0.91 | 0.67 | 1.26 | 0.5764 | 73.7% |  | 0.79 | 0.46 | 1.34 | 0.3803 | 26.3% |
| PC ae C34:0 | 1.03 | 0.80 | 1.32 | 0.9943 |  | 1.16 | 0.86 | 1.55 | 0.3323 | 75.5% |  | 0.71 | 0.43 | 1.19 | 0.1970 | 24.5% |
| PC ae C34:1 | 1.00 | 0.77 | 1.29 | 0.9943 |  | 1.08 | 0.81 | 1.44 | 0.6054 | 78.5% |  | 0.75 | 0.43 | 1.30 | 0.3069 | 21.5% |
| PC ae C34:2 | 0.93 | 0.72 | 1.22 | 0.9943 |  | 1.02 | 0.75 | 1.38 | 0.9033 | 76.7% |  | 0.70 | 0.40 | 1.21 | 0.1998 | 23.3% |
| PC ae C34:3 | 0.92 | 0.69 | 1.21 | 0.9943 |  | 0.97 | 0.70 | 1.34 | 0.8494 | 73.7% |  | 0.78 | 0.45 | 1.35 | 0.3710 | 26.3% |
| PC ae C36:0 | 0.82 | 0.64 | 1.06 | 0.9943 |  | 0.84 | 0.61 | 1.15 | 0.2635 | 63.1% |  | 0.80 | 0.53 | 1.21 | 0.2925 | 36.9% |
| PC ae C36:1 | 1.06 | 0.82 | 1.37 | 0.9943 |  | 1.20 | 0.90 | 1.60 | 0.2207 | 77.6% |  | 0.70 | 0.41 | 1.21 | 0.2025 | 22.4% |
| PC ae C36:2 | 1.02 | 0.78 | 1.34 | 0.9943 |  | 1.05 | 0.77 | 1.43 | 0.7470 | 76.1% |  | 0.93 | 0.54 | 1.62 | 0.7993 | 23.9% |
| PC ae C36:3 | 0.88 | 0.67 | 1.16 | 0.9943 |  | 0.94 | 0.69 | 1.29 | 0.6982 | 74.9% |  | 0.73 | 0.42 | 1.25 | 0.2476 | 25.1% |
| PC ae C36:4 | 1.00 | 0.78 | 1.27 | 0.9943 |  | 1.07 | 0.80 | 1.44 | 0.6422 | 68.3% |  | 0.85 | 0.56 | 1.31 | 0.4720 | 31.7% |
| PC ae C36:5 | 0.94 | 0.74 | 1.21 | 0.9943 |  | 1.00 | 0.74 | 1.37 | 0.9826 | 63.8% |  | 0.85 | 0.56 | 1.28 | 0.4391 | 36.2% |
| PC ae C38:0 | 0.94 | 0.73 | 1.22 | 0.9943 |  | 1.01 | 0.74 | 1.38 | 0.9693 | 68.5% |  | 0.82 | 0.52 | 1.31 | 0.4044 | 31.5% |
| PC ae C38:1 | 0.99 | 0.78 | 1.27 | 0.9943 |  | 1.00 | 0.75 | 1.34 | 0.9874 | 70.1% |  | 0.98 | 0.63 | 1.53 | 0.9402 | 29.9% |
| PC ae C38:2 | 0.98 | 0.75 | 1.28 | 0.9943 |  | 0.95 | 0.70 | 1.30 | 0.7550 | 74.5% |  | 1.06 | 0.62 | 1.81 | 0.8321 | 25.5% |
| PC ae C38:3 | 1.11 | 0.86 | 1.43 | 0.9943 |  | 1.25 | 0.94 | 1.67 | 0.1291 | 76.0% |  | 0.76 | 0.45 | 1.27 | 0.2906 | 24.0% |
| PC ae C38:4 | 1.00 | 0.78 | 1.28 | 0.9943 |  | 1.06 | 0.79 | 1.42 | 0.7105 | 68.9% |  | 0.89 | 0.57 | 1.38 | 0.6082 | 31.1% |
| PC ae C38:5 | 0.96 | 0.75 | 1.23 | 0.9943 |  | 0.97 | 0.71 | 1.31 | 0.8177 | 66.1% |  | 0.94 | 0.62 | 1.44 | 0.7890 | 33.9% |
| PC ae C38:6 | 0.89 | 0.68 | 1.16 | 0.9943 |  | 0.94 | 0.68 | 1.30 | 0.6966 | 68.3% |  | 0.79 | 0.49 | 1.28 | 0.3356 | 31.7% |
| PC ae C40:1 | 0.95 | 0.75 | 1.22 | 0.9943 |  | 0.99 | 0.73 | 1.34 | 0.9606 | 66.0% |  | 0.89 | 0.58 | 1.35 | 0.5722 | 34.0% |
| PC ae C40:2 | 0.97 | 0.75 | 1.25 | 0.9943 |  | 1.05 | 0.77 | 1.42 | 0.7666 | 72.1% |  | 0.79 | 0.48 | 1.28 | 0.3297 | 27.9% |
| PC ae C40:3 | 1.01 | 0.78 | 1.31 | 0.9943 |  | 1.11 | 0.83 | 1.49 | 0.4823 | 76.0% |  | 0.76 | 0.45 | 1.28 | 0.2963 | 24.0% |
| PC ae C40:4 | 0.94 | 0.73 | 1.21 | 0.9943 |  | 0.96 | 0.71 | 1.30 | 0.7930 | 70.1% |  | 0.89 | 0.56 | 1.42 | 0.6335 | 29.9% |
| PC ae C40:5 | 0.96 | 0.74 | 1.24 | 0.9943 |  | 0.95 | 0.70 | 1.31 | 0.7688 | 68.3% |  | 0.96 | 0.60 | 1.53 | 0.8646 | 31.7% |
| PC ae C40:6 | 0.96 | 0.73 | 1.25 | 0.9943 |  | 0.99 | 0.72 | 1.36 | 0.9295 | 70.5% |  | 0.90 | 0.55 | 1.47 | 0.6758 | 29.5% |
| PC ae C42:1 | 1.15 | 0.91 | 1.45 | 0.9943 |  | 1.12 | 0.84 | 1.50 | 0.4314 | 64.3% |  | 1.19 | 0.80 | 1.75 | 0.3937 | 35.7% |
| PC ae C42:2 | 1.02 | 0.79 | 1.31 | 0.9943 |  | 1.10 | 0.82 | 1.49 | 0.5287 | 71.4% |  | 0.83 | 0.52 | 1.33 | 0.4403 | 28.6% |
| PC ae C42:3 | 0.95 | 0.74 | 1.23 | 0.9943 |  | 0.98 | 0.71 | 1.35 | 0.8960 | 65.5% |  | 0.90 | 0.58 | 1.40 | 0.6473 | 34.5% |
| PC ae C42:4 | 1.01 | 0.79 | 1.29 | 0.9943 |  | 1.06 | 0.78 | 1.42 | 0.7200 | 70.1% |  | 0.90 | 0.57 | 1.43 | 0.6670 | 29.9% |
| PC ae C42:5 | 0.93 | 0.71 | 1.22 | 0.9943 |  | 0.93 | 0.68 | 1.29 | 0.6758 | 71.2% |  | 0.92 | 0.56 | 1.52 | 0.7384 | 28.8% |
| PC ae C44:3 | 1.13 | 0.88 | 1.45 | 0.9943 |  | 1.30 | 0.97 | 1.74 | 0.0830 | 71.6% |  | 0.81 | 0.51 | 1.29 | 0.3653 | 28.4% |
| PC ae C44:4 | 0.96 | 0.74 | 1.23 | 0.9943 |  | 0.98 | 0.72 | 1.32 | 0.8870 | 69.7% |  | 0.91 | 0.58 | 1.44 | 0.6917 | 30.3% |
| PC ae C44:5 | 1.00 | 0.77 | 1.28 | 0.9943 |  | 1.01 | 0.75 | 1.36 | 0.9644 | 70.3% |  | 0.97 | 0.61 | 1.54 | 0.9015 | 29.7% |
| PC ae C44:6 | 1.06 | 0.82 | 1.37 | 0.9943 |  | 1.16 | 0.85 | 1.57 | 0.3547 | 70.0% |  | 0.87 | 0.55 | 1.39 | 0.5695 | 30.0% |
| **lyso-Phosphatidylcholines** | |  |  |  |  |  |  |  |  |  |  |  |  |  |  |  |
| lysoPC a C14:0 | 0.93 | 0.75 | 1.15 | 0.9943 |  | 1.00 | 0.79 | 1.27 | 0.9973 | 79.7% |  | 0.70 | 0.43 | 1.13 | 0.1407 | 20.3% |
| lysoPC a C16:0 | 0.90 | 0.70 | 1.15 | 0.9943 |  | 0.96 | 0.72 | 1.30 | 0.8094 | 69.1% |  | 0.77 | 0.50 | 1.20 | 0.2521 | 30.9% |
| lysoPC a C16:1 | 0.93 | 0.74 | 1.18 | 0.9943 |  | 1.05 | 0.78 | 1.42 | 0.7313 | 63.9% |  | 0.76 | 0.51 | 1.12 | 0.1646 | 36.1% |
| lysoPC a C17:0 | 1.03 | 0.80 | 1.34 | 0.9943 |  | 1.13 | 0.84 | 1.52 | 0.4161 | 74 |  | 0.80 | 0.49 | 1.33 | 0.3889 | 26 |
| lysoPC a C18:0 | 0.92 | 0.71 | 1.20 | 0.9943 |  | 0.97 | 0.71 | 1.33 | 0.8523 | 69.1 |  | 0.82 | 0.51 | 1.32 | 0.4192 | 30.9 |
| lysoPC a C18:1 | 0.90 | 0.71 | 1.15 | 0.9943 |  | 0.93 | 0.68 | 1.27 | 0.6606 | 62.7 |  | 0.85 | 0.57 | 1.27 | 0.4372 | 37.3 |
| lysoPC a C18:2 | 0.99 | 0.75 | 1.30 | 0.9943 |  | 1.05 | 0.76 | 1.45 | 0.7629 | 70.5 |  | 0.85 | 0.52 | 1.40 | 0.5292 | 29.5 |
| lysoPC a C20:3 | 1.02 | 0.80 | 1.30 | 0.9943 |  | 1.18 | 0.87 | 1.60 | 0.2810 | 65.1 |  | 0.77 | 0.51 | 1.16 | 0.2075 | 34.9 |
| lysoPC a C20:4 | 0.98 | 0.78 | 1.25 | 0.9943 |  | 1.07 | 0.80 | 1.45 | 0.6376 | 64 |  | 0.84 | 0.57 | 1.25 | 0.4002 | 36 |
| lysoPC a C28:1 | 1.05 | 0.83 | 1.32 | 0.9943 |  | 1.07 | 0.82 | 1.39 | 0.6207 | 75.4 |  | 1.00 | 0.63 | 1.59 | 0.9871 | 24.6 |
| **Sphingomyelins** |  |  |  |  |  |  |  |  |  |  |  |  |  |  |  |  |
| SM C16:0 | 0.95 | 0.74 | 1.22 | 0.9943 |  | 0.93 | 0.69 | 1.26 | 0.6437 | 69.4 |  | 0.99 | 0.64 | 1.56 | 0.9824 | 30.6 |
| SM C16:1 | 1.05 | 0.81 | 1.36 | 0.9943 |  | 1.06 | 0.78 | 1.43 | 0.7190 | 74.5 |  | 1.02 | 0.61 | 1.71 | 0.9415 | 25.5 |
| SM C18:0 | 0.98 | 0.76 | 1.26 | 0.9943 |  | 0.94 | 0.69 | 1.28 | 0.7107 | 66.2 |  | 1.05 | 0.69 | 1.62 | 0.8131 | 33.8 |
| SM C18:1 | 0.99 | 0.75 | 1.29 | 0.9943 |  | 0.91 | 0.66 | 1.26 | 0.5727 | 69.2 |  | 1.17 | 0.73 | 1.90 | 0.5141 | 30.8 |
| SM C20:2 | 0.90 | 0.67 | 1.21 | 0.9943 |  | 0.81 | 0.56 | 1.18 | 0.2757 | 63.1% |  | 1.06 | 0.65 | 1.73 | 0.8140 | 36.9% |
| SM C24:0 | 0.90 | 0.67 | 1.21 | 0.9943 |  | 1.08 | 0.81 | 1.44 | 0.6147 | 69.7% |  | 0.72 | 0.46 | 1.11 | 0.1388 | 30.3% |
| SM C24:1 | 0.95 | 0.75 | 1.21 | 0.9943 |  | 0.85 | 0.62 | 1.16 | 0.3075 | 66.2% |  | 1.01 | 0.65 | 1.56 | 0.9617 | 33.8% |
| SM C26:1 | 0.90 | 0.70 | 1.16 | 0.9943 |  | 0.98 | 0.71 | 1.34 | 0.8888 | 66.5% |  | 0.83 | 0.53 | 1.29 | 0.4101 | 33.5% |
| SM (OH) C14:1 | 0.93 | 0.72 | 1.20 | 0.9943 |  | 1.18 | 0.89 | 1.57 | 0.2455 | 78.0% |  | 0.73 | 0.43 | 1.24 | 0.2456 | 22.0% |
| SM (OH) C16:1 | 1.06 | 0.83 | 1.37 | 0.9943 |  | 1.03 | 0.76 | 1.38 | 0.8668 | 72.4% |  | 0.93 | 0.57 | 1.50 | 0.7532 | 27.6% |
| SM (OH) C22:1 | 1.00 | 0.78 | 1.28 | 0.9943 |  | 1.09 | 0.82 | 1.47 | 0.5464 | 74.6% |  | 0.65 | 0.39 | 1.08 | 0.0942 | 25.4% |
| SM (OH) C22:2 | 0.96 | 0.74 | 1.23 | 0.9943 |  | 1.07 | 0.79 | 1.46 | 0.6519 | 77.1% |  | 0.69 | 0.39 | 1.22 | 0.2048 | 22.9% |
| SM (OH) C24:1 | 0.97 | 0.74 | 1.27 | 0.9943 |  | 1.19 | 0.89 | 1.59 | 0.2454 | 75.0% |  | 0.62 | 0.38 | 1.03 | 0.0636 | 25.0% |

a, acyl; AC, acylcarnitines; e, alkyl; LCL, lower 95% confidence limit; OR, odds ratio; PC, phosphatidylcholines; SM, sphingomyelin; UCL, upper 95% confidence limit
